# Characteristic Features of Deep Brain Lymphatic Vessels and Their Regulation by Chronic Stress

**DOI:** 10.34133/research.0120

**Published:** 2023-04-13

**Authors:** Junzhuang Chang, Bingqing Guo, Yan Gao, Wei Li, Xiaoyu Tong, Yi Feng, Nashat Abumaria

**Affiliations:** ^1^ State Key Laboratory of Medical Neurobiology and MOE Frontiers Center for Brain Science, Institutes of Brain Science, Fudan University, Shanghai 200032, China.; ^2^ Department of Integrative Medicine and Neurobiology, School of Basic Medical Sciences; Institutes of Brain Science, Brain Science Collaborative Innovation Center, State Key Laboratory of Medical Neurobiology, Institute of Acupuncture and Moxibustion, Fudan Institutes of Integrative Medicine, Fudan University, Shanghai 200032, China.

## Abstract

Studies have demonstrated that a functional network of meningeal lymphatic vessels exists in the brain. However, it is unknown whether lymphatic vessels could also extend deep into the brain parenchyma and whether the vessels could be regulated by stressful life events. We used tissue clearing techniques, immunostaining, light-sheet whole-brain imaging, confocal imaging in thick brain sections and flow cytometry to demonstrate the existence of lymphatic vessels deep in the brain parenchyma. Chronic unpredictable mild stress or chronic corticosterone treatment was used to examine the regulation of brain lymphatic vessels by stressful events. Western blotting and coimmunoprecipitation were used to provide mechanistic insights. We demonstrated the existence of lymphatic vessels deep in the brain parenchyma and characterized their features in the cortex, cerebellum, hippocampus, midbrain, and brainstem. Furthermore, we showed that deep brain lymphatic vessels can be regulated by stressful life events. Chronic stress reduced the length and areas of lymphatic vessels in the hippocampus and thalamus but increased the diameter of lymphatic vessels in the amygdala. No changes were observed in prefrontal cortex, lateral habenula, or dorsal raphe nucleus. Chronic corticosterone treatment reduced lymphatic endothelial cell markers in the hippocampus. Mechanistically, chronic stress might reduce hippocampal lymphatic vessels by down-regulating vascular endothelial growth factor C receptors and up-regulating vascular endothelial growth factor C neutralization mechanisms. Our results provide new insights into the characteristic features of deep brain lymphatic vessels, as well as their regulation by stressful life events.

## Introduction

Lymphatic vessels play a key role in waste clearance, fluid transport, and immune surveillance in the periphery [1]. In the brain, a glial lymphatic (glymphatic) pathway was identified. The glymphatic path exchanges cerebrospinal fluid and interstitial fluid through the spaces between blood endothelial cells and astrocytes [2]. A network of lymphatic vessels was later discovered in the meninges of the mouse brain [3]. Follow-up studies demonstrated that such a meningeal lymphatic network also exists in nonhuman primates and humans [4]. Functionally, the meningeal lymphatic vessels drain macromolecules, waste, and immune cells from the cerebrospinal fluid to cervical lymph nodes in the peripheral lymphatic system [3,5]. Developmentally, meningeal lymphatic vessels growth is modulated by vascular endothelial growth factor C (VEGF-C) signaling pathway [5]. Evidence suggest that meningeal lymphatic vessels can be regulated during certain brain pathologies including aging [6,7], Alzheimer’s disease [7], Parkinson’s disease [8], and/or traumatic brain injuries [9]. Although a study on whole-mount immunofluorescence preparations of the skull and brain reported that no lymphatic vessels were observed on pia mater and brain parenchyma [10], it remains unknown whether lymphatic vessels could extend deep into brain tissues. If so, it is also unknown whether deep brain lymphatic vessels are rigid or can dynamically change in response to external environmental factors.

Stressful life events are part of individuals’ daily lives. Stress triggers physiological responses in an organism’s body, such as increases in stress hormones, changes in inflammatory mediators, and elevations in cytokines [11]. Therefore, chronic stress has been linked to a range of diseases, including cardiovascular diseases, cancer, anxiety, depression, and immune system dysfunctions [12]. Chronic stress also reduces the circulation of the glymphatic system in the brain [13] and causes remodeling of the lymphatic vessels inside breast cancer tumors [14]. Thus, we hypothesized that chronic stress might be one of the major external environmental factors regulating deep brain lymphatic vessels. In the current study, we used tissue clearing techniques, fluorescent immunostaining, brain imaging, and flow cytometry to determine the presence of deep brain lymphatic vessels and characterize them. We also studied how chronic stress affects these vessels.

## Results

### Detection of lymphatic vessels deep in the mouse brain

**Fig. 1.**
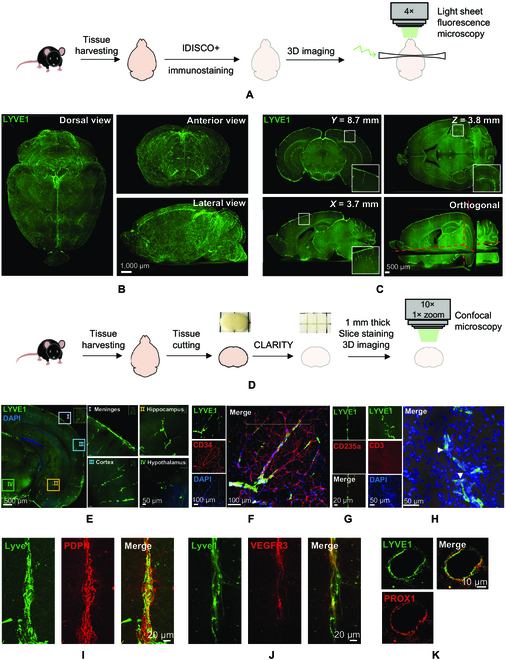
Lymphatic vessels exist deep in the mouse brain. (A) Schematic representation of experimental design of iDISCO+ tissue clearing procedure, immunostaining, and whole-brain imaging by light-sheet microscopy. (B) Light-sheet microscope fluorescent images of LYVE1 immunostaining of cleared whole-mount brain from different views. (C) Examples of optical sections taken from of whole-mount mouse brain from coronal (*Y*), sagittal (*X*), horizontal (*Z*), and orthogonal planes. Insets: Selected areas within the optical sections magnified to show the fluorescent signal of LYVE1 staining. (D) Schematic representation of experimental design of CLARITY tissue clearing procedure, immunostaining, and confocal microscopy imaging of thick brain sections. (E) Confocal images of LYVE1 fluorescent in cleared 1-mm-thick coronal brain section from mouse brain. Selected areas within the section magnified to show the fluorescent signal of LYVE1 staining in different areas. (F) Coimmunostaining of LYVE1 and CD34 in 1-mm coronal section. (G) Coimmunostaining of LYVE1 and CD235a in 16-μm brain section. (H) Same as (F) but coimmunostaining of LYVE1 and CD3. White arrows indicate T cells detected within the deep brain lymphatic vessels. (I) Coimmunostaining of LYVE1 and podoplanin (PDPN) in 16-μm brain section. (J) Same as (I) but coimmunostaining of LYVE1 and VEGFR3. (K) Same as (I) but coimmunostaining of LYVE1 and PROX1. DAPI, 4′,6-diamidino-2-phenylindole.

LYVE1 is a major marker of mature lymphatic vessels [15]. Tissue clearing methods, on the other hand, facilitate investigations of the spatial characteristics of certain structures inside opaque tissues. Therefore, we combined iDISCO+ (immunolabeling-enabled three-dimensional imaging of solvent-cleared organs) tissue clearing [16], LYVE1 immunostaining, and light-sheet whole-brain imaging to detect the lymphatic vessels deep in the brain and studied their characteristics and distribution in different brain regions (Fig. 1A). We first verified the specificity of the LYVE1 antibody used in the current study by using Western blotting and coimmunoprecipitation (Fig. S1A and B). The 3-dimensional (3D) reconstruction of the mouse brain showed LYVE1-positive signals that were consistently detected on the meninges of the brain, especially around the superior sagittal sinus area (Fig. 1B and Movie S1). LYVE1 signals were not present in the ventricles (Fig. S1C). Optical sectioning of the whole brain revealed that the LYVE1 signal remained deep inside the brain, resembling vessel-like structures. Vessel-like structures were observed in the cortex, cerebellum, hippocampus, midbrain, and brainstem (Fig. 1C). In contrast, little, if any, signal was detected in the striatum (Fig. 1C, horizontal section).

To confirm these observations, we used CLARITY [17], immunostaining, and confocal microscopy imaging in thick brain sections (Fig. 1D). Again, the LYVE1 signal, resembling vessel-like structures, was detected not only in meningeal areas but also in the cortex, hippocampus, and midbrain (Fig. 1E). We examined whether the observed vessels in the brain were accompanied by blood vessels, as reported previously for lymphatic vessels in the periphery [18]. Coimmunostaining of CD34 (blood vessel marker) and LYVE1 revealed that the LYVE1-labeled vessel-like structures ran parallel to the blood vessels and that the branching of LYVE1-stained vessels was less extensive than that of the blood vessels (Fig. 1F and Movie S2). The CD34 labeled blood vessels network was more complex and extensive than the LYVE1-labeled vessel-like network (Movie S2). Coimmunostaining of LYVE1 and the red blood cells marker CD235a revealed no colocalization of the signals in the brain (Fig. 1G), indicating that the LYVE1-labeled vessels do not have red blood cells inside them. Coimmunostaining of CD3 (a T cell marker) and LYVE1 revealed the presence of T cells in LYVE1-labeled vessel-like structures (Fig. 1H). Finally, we also found that LYVE1 signal in the identified brain vessels colocalized with 3 other lymphatic endothelial cell markers including podoplanin, vascular endothelial growth factor receptor 3 (VEGFR3), and the transcription factor PROX1 [19] (Fig. 1I to K). Thus, functional lymphatic vessels could be detected not only within meningeal structures on the brain but also deep in the brain.

### Characteristics and distribution of deep brain lymphatic vessels

**Fig. 2.**
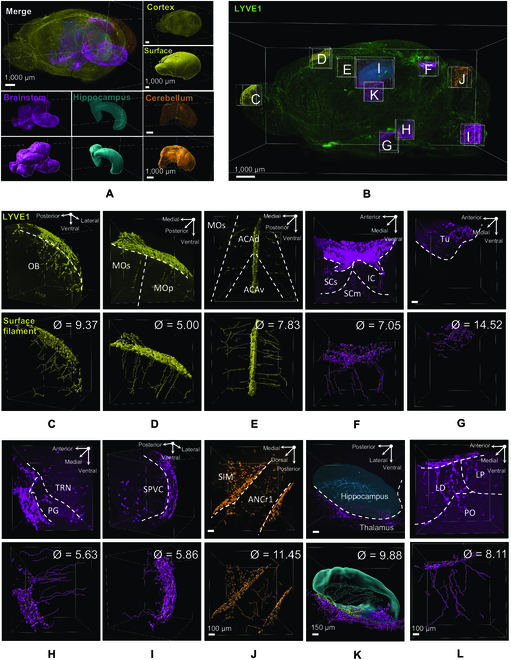
Characteristics of deep brain lymphatic vessels. (A) and (B) 3D reconstruction of whole brain with 3D reconstruction of the selected and analyzed brain regions. Different brain regions highlighted in (A) and (B) are color-coded throughout all the images below. (C to L) Top: Original LYVE1 signals. Bottom: IMARIS processed images by using Filament and Surface algorithm showing the origin of the deep brain lymphatic vessels in each brain region and their branching, direction, and diameter (Ø). OB, olfactory bulb; MOs, secondary motor cortex; MOp, primary motor cortex; ACAd, anterior cingulate cortex dorsalis; ACAv, anterior cingulate cortex ventralis; LD, lateral dorsal nucleus of thalamus; LP, lateral posterior nucleus of the thalamus; PO, posterior complex of the thalamus; Tu, tuberal nucleus; PG, pontine gray; SPVC, caudal part of spinal nucleus of the trigeminal; TRN, tegmental reticular nucleus (all in brainstem); SCs, sensory superior colliculus; SCm, motor superior colliculus; IC, inferior colliculus; SIM, simple lobule (in midbrain); ANCr1, ansiform lobule crus 1 (in cerebellum).

Using the IMARIS surface algorithm, we masked the whole brain. Brain regions with the strongest and most extensive LYVE1 signals (the cortex, olfactory bulb, hippocampus, midbrain, brainstem, and cerebellum) were delineated (see Materials and Methods), their 3D structures were constructed, and their lymphatic vessels were analyzed (Fig. 2A and B, Fig. S1D, and Movie S3). We found that the average diameter of lymphatic vessels in different brain regions ranged from 5 to 14 μm (Fig. 2A to L) and that the vessels in these regions showed low branching. We then analyzed different brain regions separately and found that the lymphatic vessels within the olfactory bulb (Fig. 2C), cortex (Fig. 2D and E), and dorsal midbrain (superior and inferior colliculus, Fig. 2F) were approximately perpendicular to the lymphatic network on the outer surface of the brain, suggesting that the vessels in these areas originated from the lymphatic vessels within the meninges. Deep in the midbrain, the lymphatic network in the ventral part of the hypothalamus originated from the ventral surface of the brain and was directed up toward the dorsal part of the hypothalamus, where the density of vessels gradually declined until it was too weak to be observed (up to ~350 μm from the lower brain surface). Lymphatic vessels within this area were mixed, i.e., they were perpendicular and parallel to the pia mater (Fig. 2G). In the brainstem, the lymphatic vessels also originated from the outer surface and were perpendicular to the pia mater. Furthermore, brainstem lymphatic networks were not detected deep in the central part of the brainstem (~450 μm from the outside surface, Fig. 2H and I). In the cerebellum, we found that the lymphatic vessels were grown from the lymphatic network on the cerebellum surface, extended deep into the cerebellum, and continued parallel to the surface (Fig. 2J). In the space separating the hippocampus and midbrain, we found that the outer lymphatic network extensively supplied both regions with lymphatic vessels. Within the hippocampus, the vessels extended dorsolaterally to cover almost the entire hippocampus, with a lower network density being observed in the dentate gyrus (Fig. 2K and Movie S4). Within the thalamus, the pattern was very similar to that observed in the cortex (Fig. 2I).

### Chronic stress regulates deep brain lymphatic vessels

To investigate whether deep brain lymphatic vessels could be influenced by external environmental factors, such as stressful life events, we exposed mice to chronic unpredictable mild stress (see Materials and Methods and Table S1) and checked for changes in deep brain lymphatic vessels in different brain areas implicated in psychiatric/stress disorders. Exposure to chronic unpredictable mild stress resulted in a gradual reduction in the consumption of the sucrose solution (~16% decrease at Day 21, Fig. S2A and B). Chronically stressed mice (Stress group) also exhibited longer immobility time in the force swimming test (~14% increase, Fig. S2A and C), shorter exploration in the center of the open field (~33% decrease), and no changes in locomotor activity (Fig. S2A and D). Thus, the 3-week chronic unpredictable mild stress protocol was effective in inducing anhedonia-like, depression-like, and anxiety-like behaviors in mice. In further support of the protocol efficacy, chronic stress also resulted in gradual reductions in body weight gain and increases in blood corticosterone (~85% increase, Fig. S2E and F). Regarding the immune system, we found that the Stress group had hypotrophy of the thymus gland but not of the spleen (Fig. S2G and H; for all statistical analyses, see Tables S2 to S4).

**Fig. 3.**
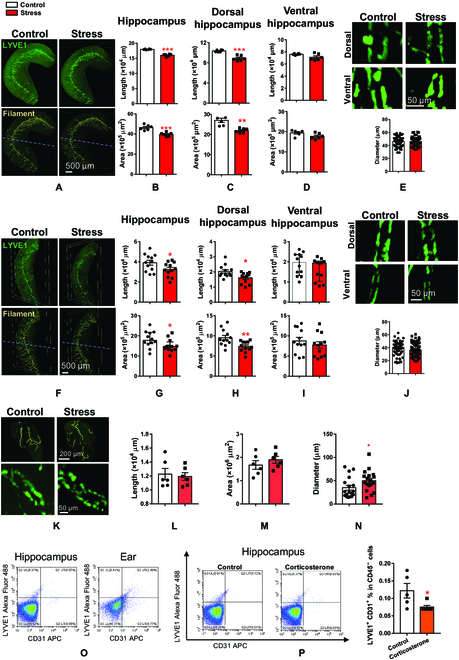
Chronic stress down-regulates deep brain lymphatic vessels. (A) Representative 3D fluorescent images of hippocampal lymphatic vessels (top) and IMARIS processed images by using *Filament* and *Surface* algorithm (bottom) taken from brains of Control and Stress groups by using iDISCO+ and light-sheet imaging in whole brain. (B to D) Quantitative analysis of lymphatic vessels length (top) and area (bottom) in whole hippocampus (B), dorsal (C), and ventral (D) parts of the hippocampus. (E) Top: Representative fluorescent images of lymphatic vessels in dorsal and ventral hippocampus of Control and Stress mice. Bottom: Quantitative analysis of lymphatic vessel diameter in the hippocampus. Data were obtained by using iDISCO+ and light-sheet imaging in whole brain. (F to J) Same as (A) to (E) but using CLARITY and confocal imaging in thick brain sections. (K) Top: Representative 3D fluorescent images of IMARIS processed images of amygdala from brains of Control and Stress groups by using iDISCO+ and light-sheet imaging in whole brain. Bottom: Representative fluorescent images of lymphatic vessels in amygdala of Control and Stress mice. (L to N) Quantitative analysis of lymphatic vessels length (L), area (M), and diameter (N) in amygdala. (O) Representative dot plots for lymphatic endothelial cells identified by CD31^+^ (endothelial cell marker) and LYVE1^+^ (lymphatic vessels marker) in the hippocampus and ear (used as positive control tissue) of adult mice. (P) Left: Representative dot plots for lymphatic endothelial cells identified by CD31^+^ and LYVE1^+^ in the hippocampus of Control or Corticosterone-treated mice. Right: Quantitative analysis of the percentage of lymphatic endothelial cells identified by CD31^+^ and LYVE1^+^ in the hippocampus of Control or Corticosterone-treated mice. Data are presented as mean ± SEM. *P < 0.05, **P < 0.01, ***P < 0.001. For detailed statistical analysis, see Tables S2 to S4.

Using iDISCO+, immunostaining, and whole-brain imaging of mice in the Control and Stress groups, we found that the length and total area of hippocampal lymphatic vessels were decreased in mice in the Stress group in comparison with mice in the Control group (~10% decrease for length and ~14% decrease for area, Fig. 3A and B and Movie S5). The dorsal and ventral hippocampus were shown to respond differently to stress [20]. Analysis of the dorsal vs. ventral hippocampus revealed that reductions in length (~14% decrease) and area (~18% decrease) occurred in the dorsal but not the ventral hippocampus (Fig. 3A, C, and D). No differences were observed in the diameter of lymphatic vessels between the mice in the Stress and Control groups (Fig. 3E). These findings were confirmed by using CLARITY and confocal imaging in brain sections of mice in the Stress and Control groups. We found reductions in length and area but not diameter in the hippocampal lymphatic vessels of mice in the Stress group (Fig. 3F to J and Movie S6). Similar to the hippocampus results, whole-brain imaging revealed that chronic stress reduced the length (~41% decrease) and area (~38% decrease) but not the diameter of lymphatic vessels in the thalamus (Fig. S3A). In the amygdala, chronic stress increased lymphatic vessels diameter (~42% increase) but not length or areas (Fig. 3K to N).

In contrast, no changes in the length, area, or diameter of lymphatic vessels were found in the medial prefrontal cortex (Fig. S3B). Because the prefrontal cortex has been implicated in psychiatric and stress disorders [21], we confirmed these results by using CLARITY and confocal imaging analysis (Fig. S3C). Whole-brain imaging analysis also showed no effects of chronic stress on the lymphatic vessels within 2 other brain areas implicated in psychiatric/stress disorders, namely, the dorsal raphe nucleus and lateral habenula [22] (Fig. S3D and E; for all statistical analyses, see Tables S2 to S4). Therefore, deep brain lymphatic vessels within the hippocampus, amygdala, and thalamus appeared to be responsive to, and could be regulated by, external environmental factors, such as repeated stressful experiences.

To further demonstrate the presence of deep brain lymphatic vessels and their regulation by stress using different approaches, we detected lymphatic vessel markers by flow cytometry and studied their regulation by chronic corticosterone treatment (alternative animal model of chronic stress) [23,24].

Using flow cytometry, we found that 2 markers of lymphatic endothelial cells, namely, LYVE1 and podoplanin, can be detected in the hippocampus as well as in the ear (used as positive control) of control mice (Fig. 3O and Fig. S4A and B). As an additional control, LYVE1 and podoplanin signals that were codetected with the macrophages marker (CD45) were excluded (Fig. 3O and Fig. S4A and B). Furthermore, similar to chronic mild stress, we found that chronic treatment with corticosterone resulted in anhedonia-/depression-/anxiety-like behavior (Fig. S4C to F). In line with our immunohistochemistry and imaging studies, we found that chronic corticosterone treatment reduced the percentage of LYVE1-positive cells in the hippocampus of treated mice in comparison with control mice (~38% decrease, Fig. 3P). Therefore, using immunohistochemistry, light-sheet microscopy, confocal microscopy, flow cytometry, multiple lymphatic vessel markers (LYVE-1, podoplanin, VEGFR3, and PROX1), and 2 different animal models of stress-like pathologies, we demonstrate that lymphatic vessels can be detected deep in brain tissue and that the vessels can be regulated by stressful life events in brain region-dependent manner.

### VEGF signaling might contribute to the regulation of deep brain lymphatic vessels by stress

Chronic stress is associated with changes in inflammation and immune systems [25] as well as growth factors [26]. On the other hand, lymphatic vessels are known to be regulated by a balance between destructive inflammatory mediators (interleukin-1β [IL-1β] and tumor necrosis factor-α [TNF-α]) and pro-growing VEGF signaling [27].

**Fig. 4.**
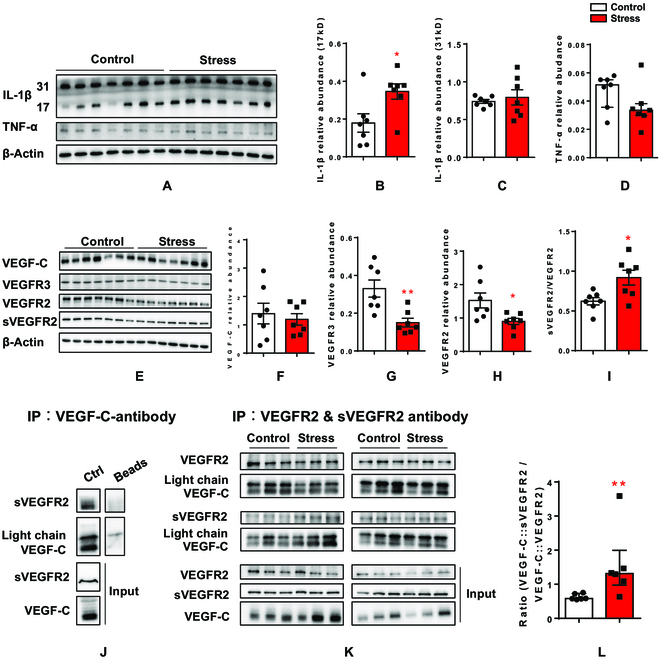
Chronic stress down-regulates VEGF-C signaling pathway. (A) Western blot images of IL-1β and TNF-α in Control and Stress groups. The codetection of β-actin bands served as loading control. (B to D) Quantitative analysis of the 17-kD IL-1β (B), 31-kD IL-1β (C), and TNF-α (D). All data were normalized to the corresponding band of the loading control β-actin. (E) Western blot images of VEGF-C, VEGFR2, and VEGFR3 as well as the cleaved and soluble VEGFR2 (sVEGFR2) in Control and Stress groups. The codetection of β-actin bands served as loading control. (F to I) Quantitative analysis of VEGF-C (F), VEGFR3 (G), VEGFR2 (H), and sVEGFR2 (I, presented as sVEGFR2/VEGFR2 ratio). All data were normalized to the corresponding band of the loading control β-actin. (J) Coimmunoprecipitation of VEGF-C and sVEGFR2 in hippocampal lysates by using VEGF-C antibody. (K) Coimmunoprecipitation of VEGF-C and VEGFR2 or sVEGFR2 in hippocampal lysates obtained from Control or Stress mice by using VEGFR2 and sVEGFR2 antibodies. (L) Quantitative analysis of the fractions of VEGF-C that binds to sVEGR2 as a ratio to that binding to VEGFR2 in Control and Stress groups. Data are presented as means ± SEM. *P < 0.05, **P < 0.01. For detailed statistical analysis, see Tables S2 to S4.

To provide mechanistic insights into how chronic stress reduced lymphatic vessels, we used quantitative Western blot analysis to first quantify inflammatory factors in the hippocampus of mice in the Control and Stress groups. We found that chronic stress up-regulated IL-1β (17 kD) but not IL-1β (31 kD) in the hippocampi of mice in the Stress group (Fig. 4A to C). Furthermore, the expression of the inflammatory factor TNF-α was not regulated by stress (Fig. 4A and D). Therefore, we concluded that destructive inflammatory processes might not be the major factor underlying the effects of stress on deep brain lymphatic vessels in the hippocampus. VEGF-C signaling plays an important role in lymphatic endothelial cell proliferation and lymphatic vessel generation [5]. Next, we quantified the expression of VEGF-C as well as VEGFR3 and VEGF receptor 2 (VEGFR2) in the hippocampus of mice in the Stress and Control groups. We found that although chronic stress did not change VEGF-C expression (Fig. 4E and F), it down-regulated the expression of VEGFR2 and VEGFR3 (Fig. 4E, G, and H).

VEGF-C can be neutralized and become ineffective by binding to a cleaved and soluble form of its receptor 2 (sVEGFR2) [28,29]. Therefore, the normal VEGF-C expression level in mice in the Stress group (Fig. 4E and F) might not mean that all VEGF-C was functional. We quantified sVEGFR2 in the hippocampus and found that chronic stress resulted in increases in the sVEGFR2/VEGFR2 ratio (~47% increase, Fig. 4I), suggesting that the cleaved and soluble form of receptor 2 was up-regulated by stress. Using coimmunoprecipitation, we found that VEGF-C can interact with sVEGFR2 in the mouse hippocampus (Fig. 4J). Using hippocampal lysates from mice in the Stress and Control groups, we combined coimmunoprecipitation with quantitative Western blotting to determine the portion of VEGF-C binding to each form of receptor 2 (nonfunctional soluble and functional nonsoluble). We found that in control hippocampal lysates, the majority of VEGF-C was bound to the functional full-length receptor, as the VEGF-C::sVEGFR2/VEGF-C::VEGFR2 ratio was below 1 (~0.6, Fig. 4K and I). This ratio was found to be higher in hippocampal lysates from mice in the Stress group (~149% increase, Fig. 4K and I; for all statistical analyses, see Tables S2 to S4), indicating that the majority of VEGF-C in the hippocampus of mice in the Stress group was neutralized and nonfunctional.

Data suggest that chronic stress might reduce hippocampal lymphatic vessels by reducing the activity of VEGF-C signaling in 2 ways: down-regulating the expression of its receptors and decreasing the amount of VEGF-C available for binding to its functional receptors.

## Discussion

In this study, we observed LYVE1-positive vessel-like structures deep inside the mouse brain parenchyma that can also be labeled by 3 other well-known markers of lymphatic vessels; namely, podoplanin [30], PROX1 [31], and VEGFR3 [32]. In support of these observations, we also detected lymphatic endothelial cell markers deep in brain tissue, suggesting the presence of lymphatic endothelial cells (necessary for forming lymphatic vessels). In line with the functional criteria used to identify lymphatic vessels in the periphery, we found that the deep brain vessels were running along with/parallel to blood vessels, carrying T cells inside and lacking blood cells. Based on all that, the vessels-like structures that we observed have the distinct transcriptional/metabolic/growth factors/membrane receptors markers as well as the typical structural and functional features of the lymphatic vessels reported before [18,19]. Therefore, we concluded that these are functional deep brain lymphatic vessels. Structural and distribution analysis revealed that majority of the deep brain lymphatic vessels originated from the outer surface of the brain. They run deep into the brain in a vertical or parallel direction to that of the pia mater except in the hippocampus, where the vessels travel inside in a dorsolateral direction. Furthermore, we found that environmental factors, such as repeated stressful experiences and/or persistent elevation of stress hormones, regulate the length, areas, or diameters of the lymphatic vessels in brain region-dependent manner. Chronic stress might regulate the lymphatic network by VEGF-C signaling.

In contradiction with the current study, imaging studies in transgenic mice and/or whole-mount immunostained preparations of skull and brain of wild-type mice failed to see lymphatic vessels on the brain parenchyma and pia mater [10]. Several procedures to visualize lymphatic vessels were described in the study. For example, some procedures involved overnight fixing followed by staining and imaging (in wild-type mice). Other imaging protocols were applied to freshly isolated tissue from transgenic mice [10]. A careful examination of the immunostaining procedures revealed major methodological differences between both studies that might explain the contradictory findings. The source, dilution, and incubation intervals of LYVE1, VEGFR3, PROX1, and podoplanin antibodies are different. Fixing, dehydrating, and staining procedures are also greatly different. We used detailed and exhaustive fixing, dehydrating, and antibody incubation protocols that span days to weeks (see Materials and Methods); meanwhile, the previous study fixed the tissue overnight before washing and staining procedures were commenced [10]. Moreover, they used whole-mount tissue staining [10]. In the current study, we used 2 different procedures of tissue clearing, whole-brain or thick-sections staining and imaging.

In the rodent brain, the structural and functional features of the meningeal lymphatic vessels were demonstrated [3]. Similar to these features, we also found a strong signal of meningeal lymphatic vessels in the superior sagittal sinus and transverse sinus. The discontinuous pattern of LYVE1 staining in the deep brain lymphatic vessels observed in the current study is in line with previous results found in dorsal meningeal lymphatic vessels [33]. Moreover, our results showed that deep brain lymphatic vessels have fewer branches, which is in line with meningeal lymphatic vessels and opposite to peripheral lymphatic vessels [3]. Finally, the diameter range of deep brain vessels reported here is similar to that of meningeal lymphatic vessels [3] but smaller than that of peripheral lymphatic vessels [34]. Thus, deep brain and meningeal lymphatic vessels have similar features, but they are distinct from peripheral lymphatic vessels. Although the current study provides detailed description of the structural characteristics of deep brain lymphatic vessels, the functional capacity of these vessels to carry lymphatic fluid and cells as well as the lymphatic fluid pathway were not addressed. Future studies should investigate such functional features.

The hippocampus, thalamus, and amygdala are among major brain regions implicated in stress disorders [35,36]. Studies show that these regions can be affected by stress in different ways. For example, chronic stress reduces dendritic network complexity and spine density in the hippocampus; meanwhile, it enhances dendritic arborization and spine formation in amygdala [37]. Stress also impairs hippocampus-dependent learning and memory but enhances the amygdala- dependent fear learning and memory [35]. On the other hand, chronic stress promotes inflammatory mechanisms and suppresses immune system activity [25]. Thus, one might expect deep brain lymphatic vessels to be regulated by stress. Indeed, we found that the lymphatic vessels were changed by chronic stress. Importantly, the stress effects appeared to be flexible in a bidirectional manner. The length and areas of lymphatic vessels in the hippocampus and thalamus were reduced. Meanwhile, although length and areas of the vessels were not changed, the diameter of lymphatic vessels in the amygdala was increased by chronic stress. Stress had no effects on other brain regions implicated in stress disorders, such as the prefrontal cortex, dorsal raphe nucleus, and lateral habenula [22,35]. Therefore, stressful life events might regulate deep brain lymphatic vessels in a brain region-dependent manner. Furthermore, our data also suggest that intrinsic features within brain regions/subregions could contribute to the stress effects on lymphatic vessels. Reductions in length and area were mainly found in the dorsal but not the ventral hippocampus. Studies show that the dorsal and ventral parts of the hippocampus have distinct features, including diverse functions, different patterns of gene expression, altered neuronal connectivity, and different responses to stress hormones [20], which might have contributed to the different effects of stress on lymphatic vessels.

Chronic stress is known to down-regulate different growth factor signaling pathways, including VEGF signaling, in the hippocampus [26]. High corticosterone treatment was shown to regulate the stability and functionality of VEGFR2 in vitro and in vivo [38]. The VEGF-C signaling pathway is a key regulator of the growth and maintenance of lymphatic vessels [5]. In the current study, we found that chronic stress down-regulated VEGF-C signaling in the hippocampus by reducing the expression of its receptors and the amount of free and functional VEGF-C. The latter effect could be mediated by increasing soluble VEGF receptor 2, which neutralizes VEGF-C efficacy [28,29]. Therefore, here, we provided some insights into the molecular mechanisms governing the remodeling of brain lymphatic vessels during stress psychopathologies. However, specific modulations of the signaling pathway in vivo to block or mimic the stress effects were not performed due to methodological challenges. Future studies should further elucidate the role of VEGF-C signaling in regulating brain lymphatic vessels. Furthermore, we cannot exclude the possibility that stress might cause lymphatic vessels remodeling by promoting other mechanisms in other brain regions such as amygdala.

Aging can impact the immune system functions [39,40], vulnerability to stress, and stress-hormones release and function [41]. Recent studies revealed that the structure and function of meningeal lymphatic vessels are impaired in aging mice [5,6]. On the other hand, fluctuations in female sex hormones have been shown to influence immune system functions [42] and stress responses [43]. Our data demonstrate the structural characteristics of deep brain lymphatic vessels and their regulation by stressful life events in adult male mice. Female and aging mice were not used to minimize the number of factors that could impact the structures and remodeling of the vessels in the brain. Further studies are required to investigate the role of aging and/or gender in regulating deep brain lymphatic vessels during stress pathologies.

Taken together, our results demonstrate the presence of lymphatic vessels in brain parenchyma and provide new insights into their characteristic features as well as their regulation by stressful life events. Future studies should address questions related to the regulation of these vessels by gender, age, and different brain pathologies. Further investigations to elucidate the role of VEGF-C signaling in regulating brain lymphatic vessels are warranted.

## Materials and Methods

### Experimental animals

Male mice (C57BL/6J, 3 months old) were purchased from Jie Si Jie for Laboratory Animals Co., Ltd. China. All mice were group-housed (4 mice/cage) with food and water ad libitum, under a 12-h:12-h reversed light:dark cycle. Behavioral experiments were performed during the dark phase under red dim light. All experiments involving animals were approved by Fudan University Committee for Animal Care and Use (license number: SYXK-2020-0032).

### Chronic unpredictable mild stress paradigm

Mice were allocated into 2 groups randomly: control group (Control, *n* = 20) and chronic unpredictable mild stress group (Stress, *n* = 26). Chronic mild stress was performed as described before [44,45]. To avoid habituation to one repeated stressor and variations in the intensity of different stressors, we combined restraint stress with other mild unpredictable stressors (such as tilting, rat odor, and humid bedding) at random time points (Table S1). Chronic mild stress routinely began in the morning and was repeated for 21 successive days. Stress mice were restrained for 2 h daily by placing them into 50-ml well-ventilated plastic tubes (3-cm internal diameter and 11.5-cm length) with ventilation holes. Control mice were handled for 5 min daily in an adjacent room and then were returned to their home cages. All mice were weighed once every 4 d (8:00 AM to 9:00 AM). One day after behavioral testing, 2 d after last stress session, mice were sacrificed and brains, adrenal glands, thymus glands, and spleen were dissected (Fig. S2). Mice brains were later randomly assigned to different types of experiments. From these 20 controls and 26 stress mice, we used *n* = 6/group for light-sheet microscope imaging, *n* = 6/group for confocal imaging, *n* = 7/group for Western blotting and coimmunoprecipitation analysis.

### Corticosterone injection paradigm

Mice were randomly assigned to 2 groups: control group (*n* = 5) and corticosterone injection group (*n* = 6). The mice received a daily injection (i.p.) of either vehicle (5% dimethyl sulfoxide [DMSO]) or 40 mg/kg corticosterone (dissolved in 5% DMSO) at 9:00 AM for 14 successive days. One day after behavioral testing, 3 d after last injection, the mice were sacrificed, and their hippocampi were precisely dissected for flow cytometry analysis. In these experiments, we only analyzed the hippocampus because it can be precisely dissected.

### Behavioral testing

#### Sucrose preference test

Sucrose preference test was performed once a week over the 3-week experimental time course. Considering the baseline test, a total of 4 sucrose preference tests were performed. The test was conducted in 2 phases: the habituation phase (24 h) and the testing phase (15 h). The first test (baseline) included 2 phases: the habituation phase (24 h) and the testing phase (15 h). The later 3 tests included test phase only. In the habituation phase, each mouse was placed in a separate cage and subsequently exposed to 2 bottles: one contains water and the other contains 1% sucrose solution. The location of bottles was switched after 12 h to prevent position bias. In the testing phase, each mouse was exposed to both bottles in similar way but only for 15 h and the bottles location was switched after 7.5 h. The water and sucrose solutions intake after test phase was measured. Sucrose preference ratio was calculated as a percentage of the total fluid intake.

#### Open-field test

Each mouse was placed in the center of a bright open-field apparatus (50-cm length × 50-cm width × 50-cm height) with a floor divided into 9 equal areas virtually. Then, the mice were allowed to explore freely for 5 min. The session was recorded with the Limelight video-tracking system and analyzed using Limelight software. Total distance (Locomotor activity) and time in center (anxiety-like behavior) were calculated automatically by the software. In order to avoid any acute effects of the last stress session on the behavior, the test was conducted 24 h after the last exposure to stressors.

#### Forced swimming test

Each mouse was placed in a vertical plexiglass cylinder (14-cm diameter × 30-cm height) containing water (21 to 23 °C) at a depth of 18 cm. The water depth was set to prevent animals from touching the bottom with their tails or hind limbs. The mice behavior was recorded for 6 min, and the duration of immobility (s) during the last 4 min was scored. Immobility was characterized by motionless floating in the water except those movements necessary to keep the nostrils above the water. In order to avoid any acute effects of the last stress session on the behavior, the test was conducted 24 h after the last exposure to stressors.

### Corticosterone measurement

After exposure to a brief stress (cold water 4 °C for 40 s), the mice were rapidly sacrificed and trunk blood was collected in a 1.5-ml tube. The blood was centrifuged for 20 min at 4,000 rpm, and the supernatants of each sample were collected and stored at −80 °C for corticosterone assays. Serum corticosterone levels were assayed with an enzyme-linked immunosorbent assay kit (Corticosterone Parameter assay kit, R&D Systems, KGE009, USA) according to the manufacturer’s instruction. Samples were diluted with Calibrator Diluent RD5-43 at dilution 1:25. Then, 150-μl diluted samples were mixed well with 150-μl Pretreatment E and incubated for 15 min at room temperature. The supernatant was collected after centrifuging and was used for analysis. After the pretreatment procedure, the reaction and measurement assay began. First, 50 μl of Corticosterone Primary Antibody Solution was added to each well and incubated for 1 h at room temperature with gentle shaking. Then, each well was washed with 400 μl of Wash Buffer 3 times. Second, 100 μl of Pretreatment Buffer was added to each well, and then 50 μl of standard, control, or each sample was added to the appropriate wells. Fifty microliters of the Corticosterone Conjugate solution was also added to all wells. After 2 h of incubation at room temperature followed by washing steps, 200 μl of Substrate Solution was added to each well. Finally, 100 μl of Stop Solution was added to each well. The optical density of each well was measured at 540 nm within 30 min using a microplate reader MultiScan Go (Thermo, USA). A standard curve was conducted. All samples were in duplicate, and the results were expressed as nanograms per milliliter.

### iDISCO+ tissue clearing

The mice were placed under deep anesthesia with 2% isoflurane and then perfused with 20 ml of phosphate-buffered saline (PBS) and 20 ml of 4% paraformaldehyde (PFA)/PBS solution. Brains were quickly harvested and fixed in 4% PFA/PBS at 4 °C overnight and then postfixed for 1 h at room temperature. Brains were stored in PBS at 4 °C for further processing. For tissue clearing, we used the iDISCO+ method as described online (https://idisco.info/) with a little modification. For pretreatment, brains were washed in PBS at room temperature 3 times, 30 min each. The brains were dehydrated at room temperature by gradient methanol aqueous solutions (20%, 40%, 60%, 80%, and 100%, 1 h each). For a final removal of any residual water, the brains were washed in 100% methanol solution for additional 2 h. After dehydrating, brains were incubated in 66% dichloromethane/methanol solution overnight at room temperature and then washed in 100% methanol for 2 h. Brains were bleached overnight at 4 °C in 5% H_2_O_2_/methanol solution and then rehydrated in a series of methanol solutions (80%, 60%, 40%, and 20%, PBS;1 h each). After PBS treatment, brains were washed in PBS-T twice (PBS-T: 0.2% Triton X-100 in PBS, 1 h each).

Once pretreatment was finished, the brains were incubated in Permeabilization solution (20% DMSO, 2.3% w/v glycine in PBS-T) for 2 d at 37 °C. Then, the brains were blocked in blocking solution (10% DMSO, 6% donkey serum in PTwH [0.2% Tween 20 and 0.01% w/v heparin in PBS]) for 2 d at 37 °C. The goat anti-LYVE1 primary antibody was dissolved in blocking solution (1:100, R&D Systems, AF2125, USA) and was used to detect lymphatic endothelial cells. The brains were incubated in primary antibodies solution for 14 d with gentle shaking (60 rpm), washed 4 to 5 times overnight, and then were incubated for 10 d with a donkey anti-goat secondary antibody (Alexa488 conjugated, 1:500, Invitrogen, A11055, USA). Finally, the brains were washed overnight, dehydrated, and then washed in 100% methanol overnight to remove residual water. Next day, brains were washed in 66% dichloromethane/methanol solution for 3 h at room temperature and 100% dichloromethane twice, each for 30 min. Finally, the brains were incubated in 100% dibenzyl ether until they looked transparent.

The transparent brains were placed in a chamber filled with dibenzyl ether and were imaged by using the LS18 light-sheet microscope (Nuohai Life Science, China). After setting the six 3D scanning sides (anterior, posterior, dorsal, ventral, left, and right) of the brain, 4× objective lens was used to scan. The 488 laser power was set at 95% to scan the whole brain. The Combine Software (Nuohai Life Science) was used to process the obtained raw images. For details on antibodies source, dilutions, and incubation procedures, see Table.

**Table.** Antibodies source, dilutions, and incubation conditions.

Antibody	Company	Catalog #	Dilution	Incubate condition	Application
Anti-LYVE1 goat antibody	R&D Systems, USA	AF2125	1:100	37°C for 14 d	iDISCO+
Anti-LYVE1 antibody	R&D Systems, USA	AF2125	1:50	37°C for 2 d	CLARITY
Anti-CD34 rabbit antibody	Abcam, USA	ab81289	1:50	37°C for 2 d	CLARITY
Anti-CD3 rabbit antibody	Abcam, USA	ab5690	1:50	37°C for 2 d	CLARITY
Anti-LYVE1 goat antibody	R&D Systems, USA	AF2125	1:200	4°C overnight	Coimmunostaining
Anti-Podoplanin mouse antibody	Cell Signaling Technology, USA	26981	1:100	4°C overnight	Coimmunostaining
Anti-VEGFR3 rabbit antibody	Arigo,China	ARG65711	1:100	4°C overnight	Coimmunostaining
Anti-PROX1 rabbit antibody	Cell Signaling Technology, USA	14963	1:50	4°C overnight	Coimmunostaining
Anti-CD235a rat antibody	BioLegend, USA	116702	1:100	4°C overnight	Coimmunostaining
Anti-CD31 rat antibody (APC)	BioLegend, USA	102409	1:100	Room temperature for 30 min	Flow cytometry
Anti-CD45 mouse antibody (eFlour450)	Invitrogen, USA	48-0459-42	1:100	Room temperature for 30 min	Flow cytometry
Anti-podoplanin mouse antibody (PE)	Invitrogen, USA	12-5381-82	1:100	Room temperature for 30 min	Flow cytometry
Anti-LYVE1 goat antibody	R&D Systems, USA	AF2125	1:100	Room temperature for 30 min	Flow cytometry
Anti-IL-1β rabbit antibody	Abcam, USA	ab9722	1:1,000	4°C overnight	WB
Anti-TNF-α rabbit antibody	Invitrogen, USA	AMC3012	1:3,000	4°C overnight	WB
Anti-VEGF-C mouse antibody	Arigo,China	ARG56212	1:1,000	4°C overnight	WB
Anti-VEGFR3 rabbit antibody	Arigo,China	ARG65711	1:1,000	4°C overnight	WB
Anti-VEGFR2 rabbit antibody	Cell Signaling Technology, USA	2479	1:1,000	4°C overnight	WB
Anti-soluble VEGFR2 rabbit antibody	Arigo,China	ARG40762	1:1,000	4°C overnight	WB
Anti-β-actin rabbit antibody	Cell Signaling Technology, USA	4970L	1:10,000	4°C overnight	WB
Anti-EGFP rabbit antibody	Proteintech, USA	50430-2-AP	1:1,000	4°C overnight	WB
Anti-VEGF-C mouse antibody	Arigo,China	ARG56212	1:100	4°C overnight	Coimmunoprecipitation
Anti-VEGFR2 rabbit antibody	Cell Signaling Technology, USA	2479	1:100	4°C overnight	Coimmunoprecipitation
Anti-soluble VEGFR2 rabbit antibody	Arigo,China	ARG40762	1:100	4°C overnight	Coimmunoprecipitation

PE, phycoerythrin; APC, allophycocyanin; WB, Western blotting; EGFP, enhanced green fluorescent protein.

### CLARITY tissue clearing

The mice were placed under deep anesthesia with 2% isoflurane, and then with 40 ml of PBS solution followed by 20 ml of 4% PFA/PBS solution. The CLARITY method we used was described before [17]. The brain was quickly harvested and postfixed in a hydrogel solution (4 g of acrylamide, 0.05 g of bis-acrylamide, 0.25 g of VA-044 initiator, and 4% PFA in PBS) at 4 °C for 3 d. The brains were then incubated at 37 °C for 3 h to polymerize and cross-link with the hydrogel matrix. The brains were cut into 1-mm-thick coronal sections. Then, the sections were postfixed in 4% PFA/PBS solution. The sections (including hippocampus region or medial prefrontal cortex region) were cleared with 4% sodium dodecyl sulfate (SDS)/PBS solution at 37 °C with gentle rotational shaking. The clearing solution was replaced daily until the tissues looked transparent (after ~3 weeks). The transparent sections were washed in PBS with 0.1% Triton X-100 at 37 °C with gentle rotational shaking for 18 h. The washing solution was replaced every 6 h to remove the residual SDS. The washed brain sections were immunolabeled with the following primary antibodies dissolved in PBS supplied with 0.1% Triton X-100 and 0.01% sodium azide at 37 °C for 48 h: anti-LYVE1 goat antibody (1:50, R&D Systems, AF2125, USA), anti-CD34 rabbit antibody (1:50, Abcam, #ab81289, USA), and anti-CD3 rabbit antibody (1:50, Abcam, #ab5690). The sections were then washed with PBS-T 6 times (each for 6 h) at 37 °C. They were subsequently immunolabeled with the corresponding secondary antibodies: donkey anti-goat (1:100, Invitrogen, #A11055) and donkey anti-Rabbit secondary antibody conjugated with Alexa568 (1:100, Invitrogen, #A10042) dissolved in PBS supplied with 0.1% Triton X-100 and 0.01% sodium azide at 37 °C for 48 h. After washing, the transparent and immune-labeled sections were embedded in 70% sorbitol solution at 37 °C until they appeared totally transparent. The embedded sections were placed in a chamber made of plasticine (1 mm thick) placed on a slide filled with sorbitol solution, and the upper part of the chamber was gently sealed using a Wellco dish (Ted Pella, #14032-120, USA) with the glass surface facing down and hence preventing the formation of small bubbles on the surface of the brain section. Images were obtained on a Nikon A1 confocal microscope. Wide-field images were obtained with 10× objective lens. After setting the edges of the brain section, scanning range (*XY* = 512 × 512 μm^2^), and the scanning depth (*Z* = maximum visible signals down to 500 μm), the sections were scanned at a speed of 0.5 μm/s, at step distance = 4 μm, possible overlap = 10%. Before Z scanning, the laser power, light gain, and offset were set using the intensity correction option of the Nikon NIS software to get the best signals. For details on antibody source, dilutions, and incubation procedures, see Table.

### 3D reconstruction and images analysis

Bitplane Imaris software 9.7 (Oxford Instruments, UK) was used to perform 3D reconstruction and 3D data analysis of the images obtained by iDISCO+ and CLARITY methods. Several algorithms in the Imaris software were used including Surface, and Filament. The Surface and Filament algorithm were used semimanually to determine the brain region morphology and to trace the lymphatic vessels (respectively). The lymphatic vessel length, diameter, and total volume were calculated by the Imaris software automatically. Relative length was quantified and normalized as length of lymphatic vessels cropped region/volume of cropped region. Relative area was quantified normalized as the total area of lymphatic vessels/volume of cropped region. For some very small brain regions such as lateral habenula and dorsal raphe nucleus, it was difficult to trace filament length and area directly. Therefore, the total fluorescent signal density was used to estimate the relative volume of lymphatic vessels in such small regions and was normalized as described above. For quantification of the lymphatic vessel diameter, we identified and sampled 3 representative vessels with the largest diameters and used them for comparing the vessel diameter between the groups. The region was determined according to the Allen Brain Atlas as reference (https://atlas.brain-map.org/atlas?atlas=602630314). The middle position of *Z* sectioning was used to separate dorsal from ventral hippocampus. The middle point of the main vertical filament was used to determine the middle *Z* position.

### Coimmunostaining in brain sections

Control naïve mice were anesthetized with 2% isoflurane and perfused with 4% PFA; brains were dehydrated by gradient sucrose solutions and then stored at −80 °C. Brain sections (16 μm) were prepared. The sections were washed with 1× PBS and incubated with blocking solution (3% donkey serum, 0.5% Triton X-100 in PBS) for 2 h at room temperature. After blocking, sections were incubated with primary antibodies in blocking solution overnight at 4 °C. Next day, sections were washed by 1× PBS and incubated with cyanine-based fluorescent (CF)-dye-conjugated secondary antibodies for 2 h at room temperature. Primary antibodies used were as follows: anti-LYVE1 goat antibody (1:200, R&D Systems, AF2125, USA), anti-podoplanin mouse antibody (1:100, Cell Signaling Technology, #26981), anti-VEGFR3 rabbit antibody (1:100, Arigo, #ARG65711), anti-PROX1 rabbit antibody (1:50, Cell Signaling Technology, #14963), and anti-CD235a rat antibody (1:100, BioLegend, #116702). CF-dye-conjugated secondary antibodies (CF488, CF555, or CF647, Biotium) were used at dilutions of 1:500. For details on antibody source, dilutions, and incubation procedures, see Table.

### Flow cytometry

Mice were anesthetized with 2% isoflurane and perfused with 1× PBS. Brains were isolated and hippocampi were dissected and digested separately. One mouse ear was chosen and used as positive-control peripheral tissue. These tissues were cut into pieces and put in digestion buffer that contained 1× Hanks’ balanced salt solution (HBSS) without Ca^2+^, Mg^2+^ (Gibco, #14175-095), 100 U/ml papain (Sangon, #9001-73-4) or 67.5 U/ml collagenase (Sangon, #9001-12-1) at 37 °C for 30 min and homogenized during the digestion. Cell suspensions were filtered over 70-μm strainers (FALCON, #352350), and filters were washed with 2 ml of 1× HBSS without Ca^2+^ and Mg^2+^. The mixture was centrifuged at 500g for 5 min. After supernatant was discarded, 5-ml 1× HBSS (Gibco, #14025-076) with 0.04% bovine serum albumin was used for resuspension the precipitation and centrifuged at 475g for 5 min. To dilute the cell suspensions, 400 μl of 1× PBS was used. From the diluted suspensions, 200 μl were used for flow cytometry. The cell dilutions were incubated with CD31 (rat antibody, APC, BioLegend, #102409), CD45 (mouse antibody, eFlour450, Invitrogen, #48-0459-42), podoplanin (mouse antibody, PE, Invitrogen, #12-5381-82) antibody, and LYVE1 primary antibodies (goat antibody, R&D Systems, AF2125, USA). For assays with LYVE-1, a donkey anti-goat Alexa488 (Invitrogen, #A11055) was used as conjugated secondary antibody. The interaction time was both limited in 30 min. The mixtures were washed and centrifuged at 500g for 5 min. The pellet was resuspended in 1× HBSS containing 2% fetal bovine serum. Samples were acquired on CytoFLEX Flow Cytometer (Beckman Coulter). Flow cytometry data were analyzed by using CytExpert software (Beckman Coulter). For details on antibodies source, dilutions, and incubation procedures, see Table.

### Western blot

Hippocampal tissue from Control and Stress groups were homogenized in radioimmunoprecipitation assay buffer (Beyotime, #P0013B, China) with protease and phosphatase inhibitors (Roche, #04906837001, #04693159001, Germany). The samples were centrifuged at 12,000 rpm at 4 °C for 15 min to obtain the supernatant (total protein). Equal amounts of proteins were resolved by 12 to 15% SDS-polyacrylamide gel electrophoresis gel and then transferred to a polyvinylidene difluoride membrane by using the BIO-RAD PowerPac Basic system. After blocking, the membranes were incubated with primary antibody overnight at 4 °C. After washing, the membranes were incubated with horseradish peroxidase-linked secondary antibodies: anti-rabbit (1:5,000, SAB, #L3032, China) or anti-mouse (1:5,000, Cell Signaling Technology, #7076s, USA) for 70 min at room temperature. The membranes were incubated with enhanced chemiluminescence solution (Tanon, #180-5001, China). The images were captured by the Tanon 5200 multi-imaging system. Blots’ images were quantified by using Gel Pro Analyzer software. The integrated optical density (IOD) of each band was measured, and the IOD of each band was normalized to the IOD of β-actin bands in the same lane. Primary antibodies used for quantitative Western blot analyses: anti-IL-1β rabbit antibody (1:1,000, Abcam, #ab9722), anti-TNF-α rabbit antibody (1:3,000, Invitrogen, #AMC3012), anti-VEGF-C mouse antibody (1:1,000, Arigo, #ARG56212, China), anti-VEGFR3 rabbit antibody (1:1,000, Arigo, #ARG65711), anti-VEGFR2 rabbit antibody (1:1,000, C-terminal, Cell Signaling Technology, #2479), anti-soluble VEGFR2 rabbit antibody (1:1,000, N-terminal, Arigo, #ARG40762) and anti-β-actin rabbit antibody (1:10,000, Cell Signaling Technology, #4970L). Western blot was also used to detect the proteins pulled out from coimmunoprecipitation. The detection procedure was performed as described above. The following primary antibodies were used for detecting the pulled out proteins: anti-VEGF-C, anti-VEGFR2, anti-soluble VEGFR2 (all same as above) and anti-enhanced green fluorescent protein rabbit antibody (1:1,000, Proteintech, #50430-2-AP). For details on antibodies source, dilutions, and incubation procedures, see Table.

### Coimmunoprecipitation

Protein lysates from Control or Stress groups were placed on ice for 30 min and centrifuged at 12,000 rpm, 4 °C for 15 min. The supernatant was collected and used for coimmunoprecipitation. Dynabeads Protein G Immunoprecipitation Kit (Invitrogen, #10007D) was used for coimmunoprecipitation of the native proteins from the brain tissue lysates. We used mouse monoclonal VEGF-C antibody (1:100, Arigo, #ARG56212) to pull down the native VEGF-C and its interacting proteins. First, 2-μl antibody was mixed with 200 μl containing1.5-mg Dynabeads and incubated at room temperature for 30 min. Then, 3-mg total proteins were mixed with Dynabeads-antibody complexes and incubated at 4 °C for 36 h. After washing, 20 μl Elution buffer, 5 μl 5× loading buffer, and 2μl β-mercaptoethanol were added into the Dynabeads-antibody-antigen complexes and the mixture was boiled at 99 °C for 5 min to elute proteins. Finally, the eluted proteins were resolved and bands were detected by Western blot. For quantifying the amount of VEGF-C that binds to VEGFR2 and to compare it with that that binds to sVEGFR2, 2 μl Rabbit Monoclonal VEGFR2 Antibody (1:100, C-terminus, Cell Signaling Technology, #2479) or 2 μl of Rabbit Polyclonal VEGFR2 antibody (1:100. N-terminal, Arigo, #ARG40762) was mixed with 200 μl containing1.5 mg Dynabeads, and incubated at room temperature for 40 min. Then, 2-mg total proteins were mixed with each Dynabeads-antibody complex and incubated at 4 °C for 40 h. After washing, 20 μl Elution buffer, 5 μl 5×loading buffer, and 2 μl β-mercaptoethanol were added into each complex and the mixtures were boiled at 99 °C for 5 min to elute proteins. Finally, the eluted proteins of both complexes were simultaneously resolved and bands were detected / analyzed by quantitative Western blot. For details on antibodies source, dilutions, and incubation procedures, see Table.

### Statistical methods

Kolmogorov-Smirnov normality test was used for detecting the data normality. F-test was used for detecting the homogeneity of variance. Two-tailed unpaired *t* test or Mann–Whitney test was used for comparisons between 2 groups, depending on the normality of data. For sucrose preference test and body weight gain data, 2-way repeated measure *ANOVA* followed Bonferroni’s post hoc test was used. All details of statistical tests, analysis and results of each figure are presented in Table S2. Results of normality test and homogeneity of variance are reported in Table S3. Data of mean, SEM, and n for all groups are reported in Table S4. Data are presented as means ± SEM. Statistical significance was defined as *P* < 0.05.

## Data Availability

Data supporting the findings of this study are available in the main text or the supplementary information. Additional data related to this paper may be requested from the authors.

## Supplementary Materials

Supplementary 1 Fig. S1. Validation experiments for the LYVE1 antibody. No LYVE1 signals in ventricles. Delineation of brain regions including LYVE1 signals deep in the brain. Fig. S2. Chronic stress effectively induced anhedonia-/depression-/anxiety-like behaviors and changes physiological readouts. Fig. S3. Effects of chronic stress on deep brain lymphatic vessels in thalamus, medial prefrontal cortex, dorsal raphe nucleus, and lateral habenula. Fig. S4. Detection of deep brain lymphatic vessels markers by flow cytometry and corticosterone injection induced depression-/anxiety-like behaviors. Table S1. Chronic unpredictable mild stress protocol. Table S2. Details of the statistical analysis of all datasets. Table S3. Results of normality and homogeneity of variance tests. Table S4. Data of mean, SEM, and N for each group.

Supplementary 2 Movie S1. LYVE1 signals in whole brain. Movie S2. LYVE1 (lymphatic vessels) and CD34 (blood vessels) signals in the mouse PFC. Movie S3. Split brain region by using Surface algorithm. Movie S4. Lymphatic vessels in hippocampus. Movie S5. Lymphatic vessels in hippocampus of Control and Stress mice (light-sheet microscopy). Movie S6. Lymphatic vessels in hippocampus of Control and Stress mice (confocal microscopy).
