# Supplementary material for: Characteristic Features of Deep Brain Lymphatic Vessels and Their Regulation by Chronic Stress
Source: Research (Wash D C). 2023 Apr 13;6:0120. doi: 10.34133/research.0120 (PMC10202180; doi:10.34133/research.0120)
Supplement: Supplementary 1 — Fig. S1. Validation experiments for the LYVE1 antibody. No LYVE1 signals in ventricles. Delineation of brain regions including LYVE1 signals deep in the brain. Fig. S2. Chronic stress effectively induced anhedonia-/depression-/anxiety-like behaviors and changes physiological readouts. Fig. S3. Effects of chronic stress on deep brain lymphatic vessels in thalamus, medial prefrontal cortex, dorsal raphe nucleus, and lateral habenula. Fig. S4. Detection of deep brain lymphatic vessels markers by flow cytometry and corticosterone injection induced depression-/anxiety-like behaviors. Table S1. Chronic unpredictable mild stress protocol. Table S2. Details of the statistical analysis of all datasets. Table S3. Results of normality and homogeneity of variance tests. Table S4. Data of mean, SEM, and N for each group. [file research.0120.f1.docx]

Characteristic Features of Deep Brain Lymphatic Vessels and Their Regulation by Chronic Stress

*Junzhuang Chang,* *Bingqing Guo, Yan Gao,* *Wei Li,* *Xiaoyu Tong,* *Yi Feng**, Nashat Abumaria*

**This PDF file includes:**

Figures S1 to S4

Tables S1 to S4

Legends for Movies S1 to S6

**Other supporting materials for this manuscript include the following:**

Movies S1 to S6

**
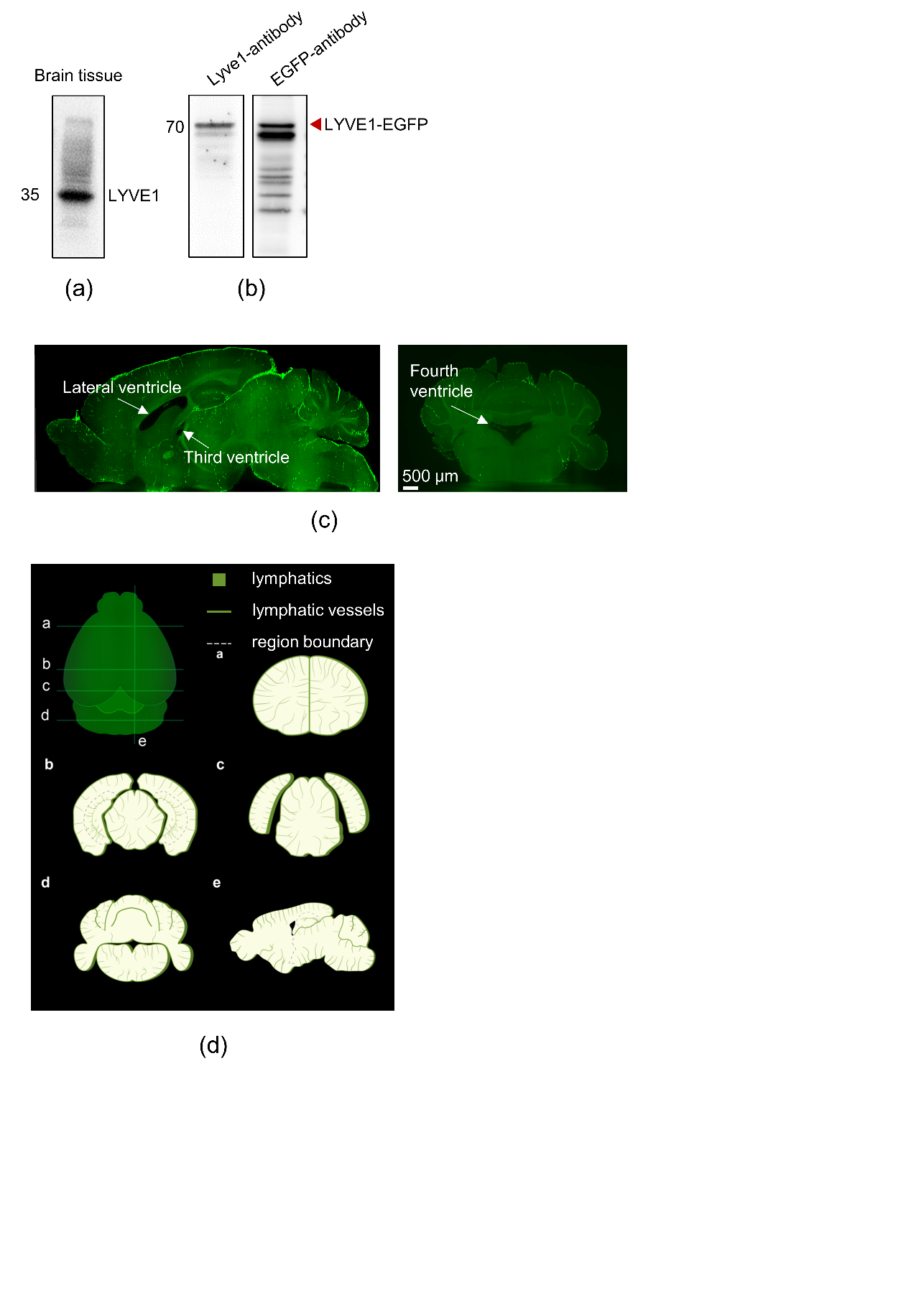
**

FIGURE S1: Validation experiments for the LYVE1 antibody. No LYVE1 signals in ventricles. Delineation of brain regions including LYVE1 signals deep in the brain. (a, b) Validation experiments for the anti-LYVE1 antibody. Western blot images of native LYVE1 in hippocampal lysates (a). Co-immunoprecipitation of LYVE1-EGFP overexpressed in 293T cell by using the LYVE1 antibody, and then detecting it by using LYVE1 antibody or the EGFP antibody. Data suggest that the antibody is able to specifically detect the LYVE1. (c) Examples of optical sections taken from of whole-mount mouse brain to showing no LYVE1 signals in the ventricles inner side. (d) Schematic representation of the LYVE1 signals in different brain regions.

**
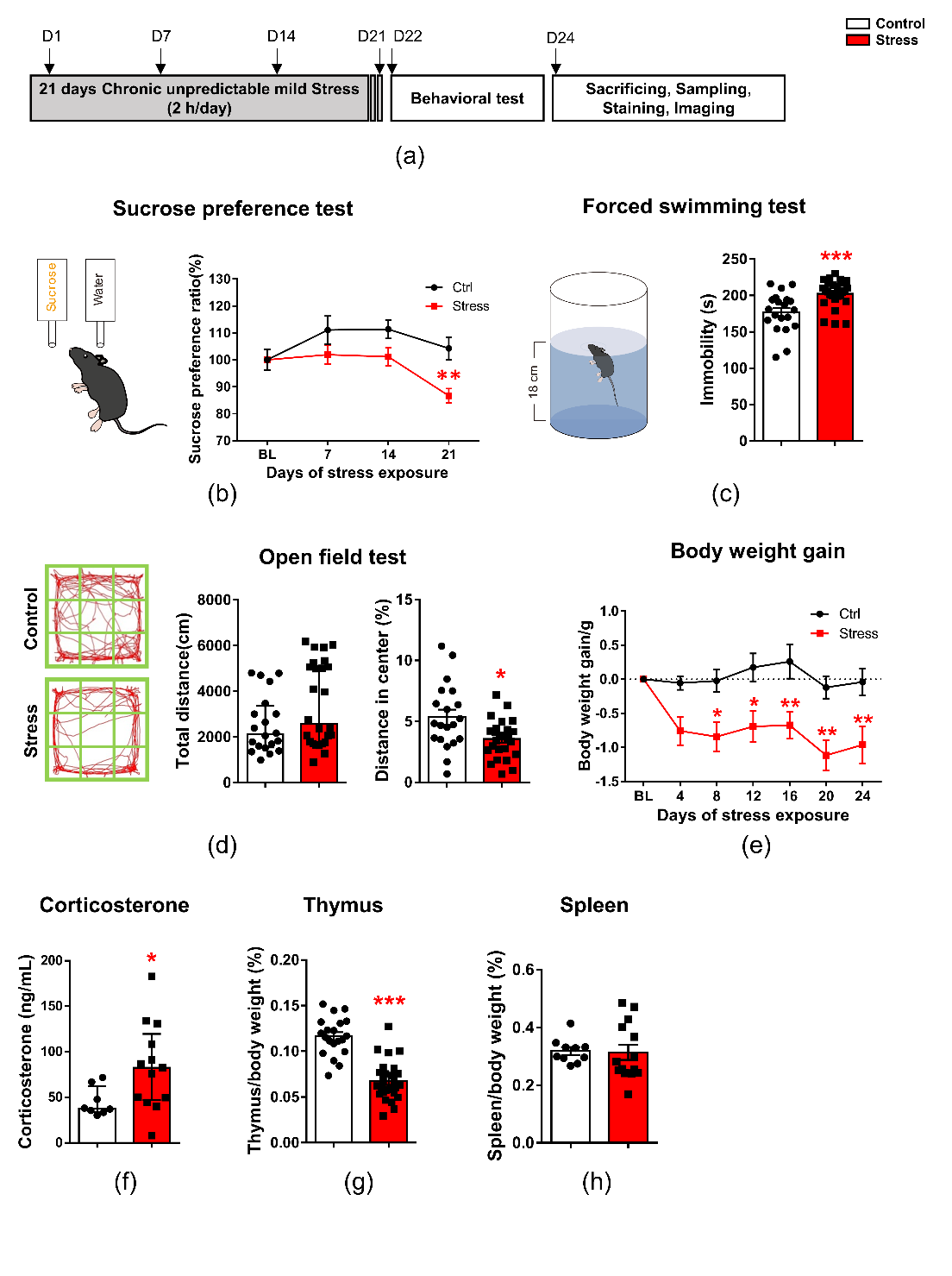
**

FIGURE S2: Chronic stress effectively induced anhedonia-/depression-/ anxiety-like behaviors, and changes physiological readouts. (a) Schematic representation of experimental design showing chronic unpredictable mild stress protocol, behavioral testing schedules, sacrificing animals and imaging. (b) Quantitative analysis of sucrose solution consumption calculated as percentage (%) of total fluid intake in Control and Stress groups over three week. (c) Quantitative analysis of immobility time of Control and Stress mice during the forced swimming test (last 4 min were analyzed). (d) Quantitative analysis of total distance(left) and distance in center (right, presented as percentage of total distance) travelled during 5 min of open field test of Control and Stress groups. (e) Quantitative analysis of body weight gain of Control and Stress groups. (f) Corticosterone concentrations in serum of Control and Stress mice quantified in collected trunk blood samples. (g, h) The thymus (g) and spleen (h) weights calculated as percentage (%) of body weight in Control and Stress mice. Data are presented as mean ± SEM. * *p* < 0.05, ** *p* < 0.01, *** *p* < 0.001. For detailed statistical analysis see Tables S2, S3 and S4.

**
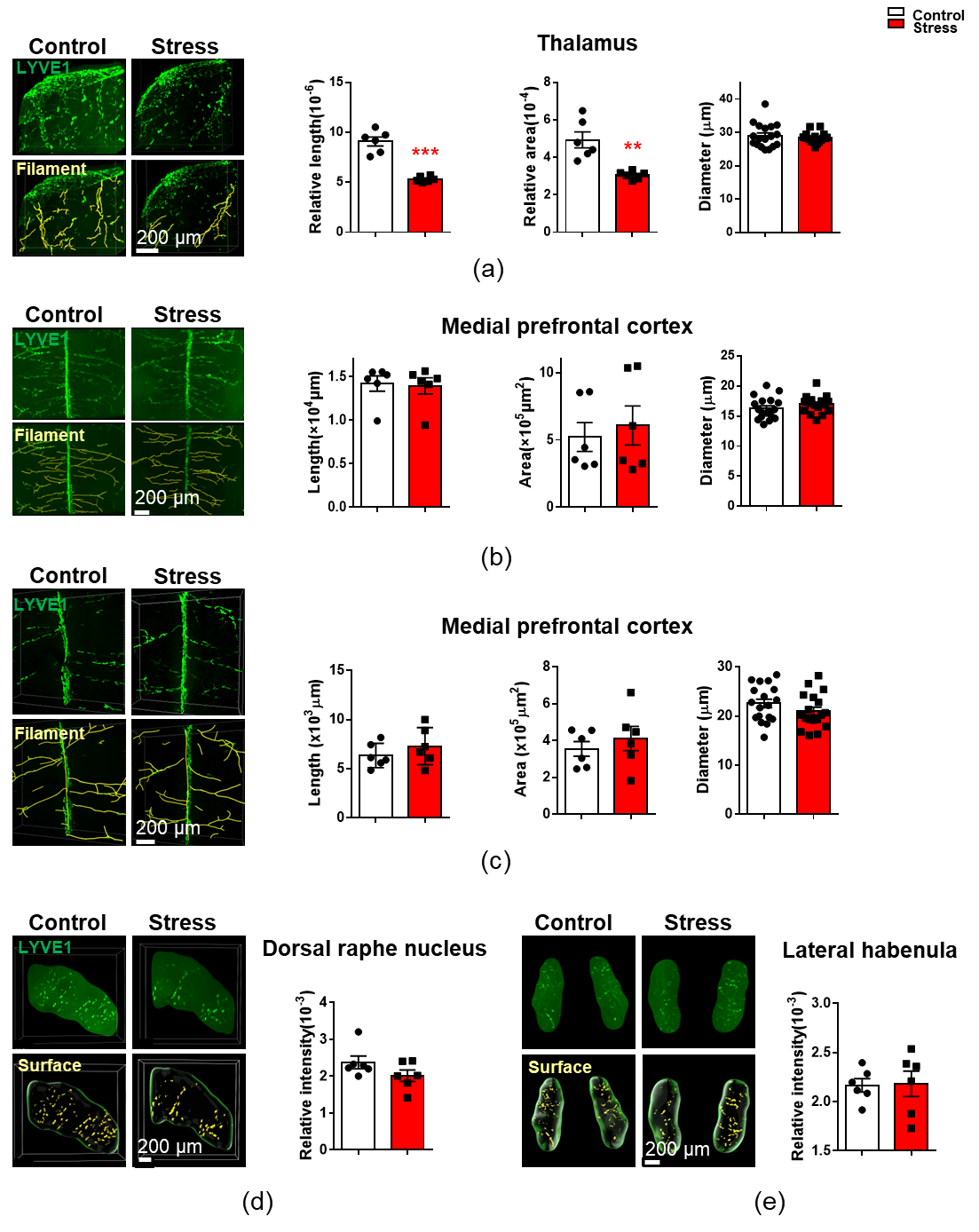
**

FIGURE S3: Effects of chronic stress on deep brain lymphatic vessels in thalamus, medial prefrontal cortex, dorsal raphe nucleus and lateral habenula. (a) Left: Representative 3D fluorescent images of thalamus deep brain lymphatic vessels (up) and IMARIS filament processed images of the vessels (down) taken from Control and Stress brains by using iDISCO+ and light sheet imaging in whole brain. Right: Quantitative analysis of lymphatic vessels length, area, and diameter within the thalamus of Control and Stress mice. (b) Same as a but in medial prefrontal cortex. (c) Same as b but using CLARITY and confocal imaging in thick brain sections. (d, e) Sam as a but showing no changes in lymphatic vessels overall intensity within dorsal raphe nucleus (d) and lateral habenula (e). Data are presented as mean ± SEM. * *p* < 0.05, ** *p* < 0.01. For detailed statistical analysis see Tables S2, S3 and S4.

**
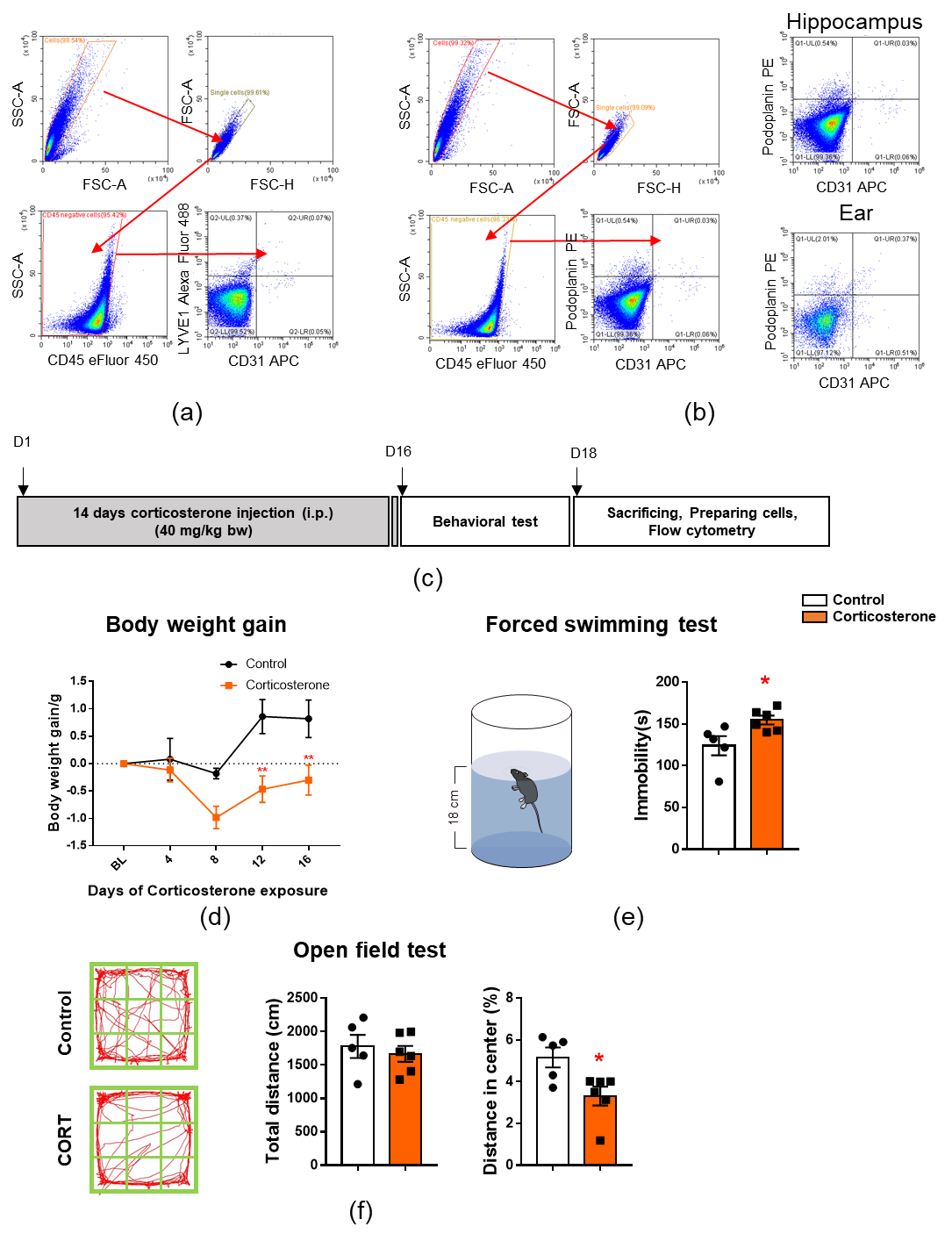
**

FIGURE S4: Detection of deep brain lymphatic vessels markers by flow cytometry and corticosterone injection induced depression-/ anxiety-like behaviors. (a) Gating strategy employed to identify lymphatic endothelial cells (endothelial cells marker: CD31^+^; lymphatic vessels marker: LYVE1^+^). (b) Left: Gating strategy employed to identify lymphatic endothelial cells using another marker (podoplanin^+^). Right: Representative dot plots for lymphatic endothelial cells identified by CD31^+^ and podoplanin^+^ in the hippocampus and ear (used as positive control tissue) of adult mice. (c) Schematic representation of experimental design showing corticosterone injection protocol, behavioral testing schedules, sacrificing animals and flow cytometry. (d) Quantitative analysis of body weight gain of Control and Corticosterone injected mice. (e) Quantitative analysis of immobility time of Control and Corticosterone groups during the forced swimming test (last 4 min were analyzed). (f) Quantitative analysis of total distance (left) and distance in center (right, presented as percentage of total distance) travelled during 5 min of open field test of the same groups of mice. Data are presented as mean ± SEM. * *p* < 0.05. For detailed statistical analysis see Tables S2, S3 and S4.

**Table S1.** Chronic unpredictable mild stress protocol

| **Day** | **Stressors** | **Time schedule** |
| --- | --- | --- |
| 1 | restraint 2h | 10:00-12:00 |
| 2 | restraint 2h with tilting 45° | 12:00-14:00 |
| 3 | restraint 2h with rat odor in cotton | 9:00-11:00 |
| 4 | restraint 2h after overnight humid bedding | 11:00-13:00 |
| 5 | restraint 2h with rat odor in cotton | 12:00-14:00 |
| 6 | restraint 2h | 9:00-11:00 |
| 7 | restraint 2h with tilting 45° | 11:00-13:00 |
| 8 | restraint 2h after overnight humid bedding | 10:00-12:00 |
| 9 | restraint 2h with tilting 45° | 9:00-11:00 |
| 10 | restraint 2h | 11:00-13:00 |
| 11 | restraint 2h after overnight humid bedding | 12:00-14:00 |
| 12 | restraint 2h with rat odor in cotton | 10:00-12:00 |
| 13 | restraint 2h after overnight humid bedding | 11:00-13:00 |
| 14 | restraint 2h with tilting 45° | 10:00-12:00 |
| 15 | restraint 2h | 12:00-14:00 |
| 16 | restraint 2h with rat odor in cotton | 9:00-11:00 |
| 17 | restraint 2h | 10:00-14:00 |
| 18 | restraint 2h after overnight humid bedding | 12:00-14:00 |
| 19 | restraint 2h with rat odor in cotton | 11:00-13:00 |
| 20 | restraint 2h with tilting 45° | 9:00-11:00 |
| 21 | restraint 2h | 12:00-14:00 |

**Table S2.** Details of the statistical analysis of all datasets.

| **Figures.** | | **Name of Test** | **n** | **Statistic value** | ***p*-value** |
| --- | --- | --- | --- | --- | --- |
| Figure. 3b | length | Two-tailed unpaired *t*- test | 6 | *Welch-corrected t* _6.048_ = 12.09 | **** p* < 0.0001 |
|  | area | Two-tailed unpaired *t*- test | 6 | *t* _10_ = 5.055 | **** p* = 0.0005 |
| Figure. 3c | length | Two-tailed unpaired *t*- test | 6 | *t* _10_ = 6.258 | **** p* < 0.0001 |
|  | area | Two-tailed unpaired *t*- test | 6 | *t* _10_ = 4.526 | *** p* = 0.0011 |
| Figure. 3d | length | Two-tailed unpaired *t*- test | 6 | *Welch-corrected t* _5.585_ = 2.132 | *p* = 0.0804 |
|  | area | Two-tailed unpaired *t*- test | 6 | *t* _10_ = 2.024 | *p* = 0.0705 |
| Figure. 3e |  | Two-tailed unpaired *t*- test | 72 | *t* _142_ = 0.05851 | *p* = 0.9534 |
| Figure. 3g | length | Two-tailed unpaired *t*- test | 12 (left and right hippocampi from 6 mice) | *t* _22_ = 2.091 | ** p* = 0.0483 |
|  | area | Mann Whitney test | 12 (same as above) | *U* = 36 | ** p* = 0.0372 |
| Figure. 3h | length | Two-tailed unpaired *t*- test | 12 (same as above) | *t* _22_ = 2.696 | ** p* = 0.0132 |
|  | area | Two-tailed unpaired *t*- test | 12 (same as above) | *t* _22_ = 3.064 | *** p* = 0.0057 |
| Figure. 3i | length | Mann Whitney test | 12 (same as above) | *U* = 50.50 | *p* = 0.2244 |
|  | area | Two-tailed unpaired *t*- test | 12 (same as above) | *t* _22_ = 0.8581 | *p* = 0.4001 |
| Figure. 3j |  | Mann Whitney test | 72 | *U* = 2243 | *p* = 0.1639 |
| Figure. 3l |  | Two-tailed unpaired *t*- test | 6 | *t* _10_ = 0.3462 | *p* = 0.7363 |
| Figure. 3m |  | Two-tailed unpaired *t*- test | 6 | *t* _10_ = 0.936 | *p* = 0.3713 |
| Figure. 3n |  | Mann Whitney test | 18 | *U* = 97 | ** p* = 0.0402 |
| Figure. 3p |  | Mann Whitney test | n1=5  n2=6 | *U* = 4.5 | *** p* = 0.0411 |
| Figure. 4b |  | Two-tailed unpaired *t*- test | 7 | *t* _12_ = 2.622 | ** p* = 0.0223 |
| Figure. 4c |  | Two-tailed unpaired *t*- test with Welch’s correction | 7 | *Welch-corrected t* _6.735_ = 0.5103 | *p* = 0.6261 |
| Figure. 4d |  | Mann Whitney test | 7 | *U* = 13 | *p* = 0.1638 |
| Figure. 4f |  | Two-tailed unpaired *t*- test | 7 | *t* _12_ = 0.4931 | *p* = 0.6308 |
| Figure. 4g |  | Two-tailed unpaired *t*- test | 7 | *t* _12_ = 3.558 | *** p* = 0.0039 |
| Figure. 4h |  | Two-tailed unpaired *t*- test with Welch’s correction | 7 | *Welch-corrected t* _8_ = 2.614 | ** p* = 0.0309 |
| Figure. 4i |  | Two-tailed unpaired *t*- test | 7 | *t* _12_ = 2.837 | ** p* = 0.0150 |
| Figure. 4l |  | Mann Whitney test | 6 | *U* = 2 | *** p* = 0.0087 |
| Figure S2b |  | Two-way ANOVA | n1=10  n2=14 | *F _(1,22)_* = 5.620 | ** p* = 0.0269 |
|  | Day 21 | Bonferroni's multiple comparisons test |  |  | *** p* = 0.0060 |
| Figure S2c |  | Two-tailed unpaired *t*- test | n1=20  n2=26 | *t* _44_ = 3.747 | **** p* = 0.0005 |
| Figure S2d | Left | Mann Whitney test | n1=20  n2=26 | *U* = 180 | *p* = 0.0773 |
|  | right | Two-tailed unpaired *t*- test with Welch’s correction | n1=20  n2=26 | *Welch-corrected t* _28.54_ = 2.671 | ** p* = 0.0124 |
| Figure S2e |  | Two-way ANOVA | n1=20  n2=26 | *F _(1,44)_* =11.83 | *** p* = 0.0013 |
|  | 8 | Bonferroni's multiple comparisons test |  |  | ** p* = 0.0295 |
|  | 12 | Bonferroni's multiple comparisons test |  |  | ** p* = 0.0169 |
|  | 16 | Bonferroni's multiple comparisons test |  |  | *** p* = 0.0078 |
|  | 20 | Bonferroni's multiple comparisons test |  |  | *** p* = 0.0036 |
|  | 24 | Bonferroni's multiple comparisons test |  |  | *** p* = 0.0089 |
| Figure S2f |  | Mann Whitney test | n1=8  n2=13 | *U* = 18 | ** p* = 0.0126 |
| Figure S2g |  | Two-tailed unpaired *t*- test | n1=20  n2=26 | *t* _44_ = 7.64 | **** p* < 0.0001 |
| Figure S2h |  | Two-tailed unpaired *t*- test with Welch’s correction | n1=10  n2=14 | *Welch-corrected t* _18.88_ = 0.1623 | *p* = 0.8728 |
| Figure S3a | Relative length | Two-tailed unpaired *t*- test | 6 | *t* _10_ =8.098 | **** p* < 0.0001 |
|  | Relative area | Two-tailed unpaired *t*- test | 6 | *t* _10_ =4.337 | *** p* = 0.0015 |
|  | Diameter | Two-tailed unpaired *t*- test with Welch’s correction | 18 | *Welch-corrected t* _23.31_ = 0.7320 | *p* = 0.4715 |
| Figure S3b | Length | Mann Whitney test | 6 | *U* = 16 | *p* = 0.8182 |
|  | Area | Mann Whitney test | 6 | *U* = 16 | *p* = 0.8182 |
|  | Diameter | Two-tailed unpaired *t*- test | 18 | *t* _34_ = 1.262 | *p* = 0.2154 |
| Figure S3c | Length | Two-tailed unpaired *t*- test | 6 | *t* _10_ = 1.035 | *p* = 0.3250 |
|  | Area | Two-tailed unpaired *t*- test | 6 | *t* _10_ = 0.7373 | *p* = 0.4779 |
|  | Diameter | Two-tailed unpaired *t*- test | 18 | *t* _34_ = 1.316 | *p* = 0.1971 |
| Figure S3d |  | Mann Whitney test | 6 | *U* = 12 | *p* = 0.3939 |
| Figure S3e |  | Two-tailed unpaired *t*- test | 6 | *t* _10_ = 0.1136 | *p* = 0.9118 |
| Figure S4d |  | Two-way ANOVA | n1=5  n2=6 | *F _(1,9)_* =6.526 | ** p* = 0.0310 |
|  | 12 | Bonferroni's multiple comparisons test |  |  | *** p* = 0.0014 |
|  | 16 | Bonferroni's multiple comparisons test |  |  | *** p* = 0.0090 |
| Figure S4e |  | Two-tailed unpaired *t*- test | n1=5  n2=6 | *t* _9_ = 2.576 | ** p* = 0.0299 |
| Figure S4f | Left | Two-tailed unpaired *t*- test | n1=5  n2=6 | *t* _9_ = 0.5355 | *p* = 0.6053 |
|  | Right | Two-tailed unpaired *t*- test | n1=5  n2=6 | *t* _9_ = 2.806 | ** p* = 0.0205 |

n1: numbers in control group.

n2: numbers in stress group.

*** *p* < 0.05, ** *p* < 0.01, *** *p* < 0.001.**

**Table S3.** Results of normality and homogeneity of variance tests.

*p* < 0.05 indicates that data do not meet normality or homogeneity of variance. * *p* < 0.05, ** *p* < 0.01.

| **Figures.** | **KS normality test** | | **F test** | |
| --- | --- | --- | --- | --- |
|  | **Group name and *p*-value** | | ***F* values** | ***p* values** |
| Figure. 3b length | Control | Stress | *F _(5,5)_* = 9.437 | ** p = 0.0278* |
|  | *p* > 0.1000 | *p* > 0.1000 |  |  |
| Figure. 3b area | Control | Stress | *F _(5,5)_* = 1.833 | *p = 0.5221* |
|  | *p* > 0.1000 | *p* > 0.1000 |  |  |
| Figure. 3c length | Control | Stress | *F _(5,5)_* = 5.639 | *p = 0.0807* |
|  | *p* > 0.1000 | *p* > 0.1000 |  |  |
| Figure. 3c area | Control | Stress | *F _(5,5)_* =4.73 | *p = 0.1133* |
|  | *p* > 0.1000 | *p* > 0.1000 |  |  |
| Figure. 3d length | Control | Stress | *F _(5,5)_* =17.05 | *** p = 0.0074* |
|  | *p* > 0.1000 | *p* > 0.1000 |  |  |
| Figure. 3d area | Control | Stress | *F _(5,5)_* =1.124 | *p = 0.0705* |
|  | *p* > 0.1000 | *p* > 0.1000 |  |  |
| Figure. 3e | Control | Stress | *F _(71,71)_* =1.395 | *p = 0.1633* |
|  | *p* > 0.1000 | *p* > 0.1000 |  |  |
| Figure. 3g length | Control | Stress | *F _(11,11)_* =1.426 | *p = 0.5664* |
|  | *p* > 0.1000 | *p* > 0.1000 |  |  |
| Figure. 3g area | Control | Stress | / | / |
|  | *p* > 0.1000 | ** p* = 0.0120 |  |  |
| Figure. 3h length | Control | Stress | *F _(11,11)_* =1.426 | *p = 0.5664* |
|  | *p* = 0.1018 | *p* = 0.1132 |  |  |
| Figure. 3h area | Control | Stress | *F _(11,11)_* =2.349 | *p = 0.1723* |
|  | *p* > 0.1000 | *p* > 0.1000 |  |  |
| Figure. 3i length | Control | Stress | / | / |
|  | *p* > 0.1000 | *** p* = 0.0081 |  |  |
| Figure. 3i area | Control | Stress | *F _(11,11)_* =1.121 | *p = 0.8527* |
|  | *p* > 0.1000 | *** p* = 0.0081 |  |  |
| Figure. 3l | Control | Stress | *F _(5,5)_* = 2.188 | *p = 0.4103* |
|  | *p* = 0.0501 | *p* > 0.1000 |  |  |
| Figure. 3m | Control | Stress | *F _(5,5)_* = 1.544 | *p = 0.6453* |
|  | *p* > 0.1000 | *p* > 0.1000 |  |  |
| Figure. 3n | Control | Stress | / | / |
|  | *** p* = 0.0056 | *p* > 0.1000 |  |  |
| Figure. 3j | Control | Stress | / | / |
|  | *p* = 0.0295 | *p* > 0.1000 |  |  |
| Figure. 3p | Control | Corticosterone | / | / |
|  | *p* > 0.1000 | **** p* < 0.0001 |  |  |
| Figure. 4b | Control | Stress | *F _(6,6)_* =1.437 | *p = 0.6710* |
|  | *p* > 0.1000 | *p* = 0.1383 |  |  |
| Figure. 4c | Control | Stress | *F _(6,6)_* =16.26 | *** p =* 0.0036 |
|  | *p* > 0.1000 | *p* > 0.1000 |  |  |
| Figure. 4d | Control | Stress | / | / |
|  | ** p* = 0.0490 | *p* = 0.0772 |  |  |
| Figure. 4f | Control | Stress | *F _(6,6)_* =3.114 | *p =* 0.1927 |
|  | *p* > 0.1000 | *p* > 0.1000 |  |  |
| Figure. 4g | Control | Stress | *F _(6,6)_* =3.999 | *p =* 0.1159 |
|  | *p* > 0.1000 | *p* > 0.1000 |  |  |
| Figure. 4h | Control | Stress | *F _(6,6)_* =5.830 | ** p =* 0.0498 |
|  | *p* > 0.1000 | *p* > 0.1000 |  |  |
| Figure. 4i | Control | Stress | *F _(6,6)_* =4.177 | *p =* 0.1056 |
|  | *p* > 0.1000 | *p* > 0.1000 |  |  |
| Figure. 4l | Control | Stress | / | / |
|  | ** p* = 0.0193 | *** p* = 0.0090 |  |  |
| Figure s2c | Control | Stress | *F _(19,25)_* =2.048 | *p =* 0.0938 |
|  | *p* > 0.1000 | *p* = 0.0882 |  |  |
| Figure S2d left | Control | Stress | / | / |
|  | *p* = 0.1609 | *** p* = 0.0029 |  |  |
| Figure S2d right | Control | Stress | *F _(19,25)_* =3.008 | ** p =* 0.0108 |
|  | *p* > 0.1000 | *p* > 0.1000 |  |  |
| Figure S2f | Control | Stress | / | / |
|  | ** p* = 0.0378 | *p* > 0.1000 |  |  |
| Figure S2g | Control | Stress | *F _(9,13)_* =5.293 | ** p =* 0.0171 |
|  | *p* = 0.2293 | *p* = 0.0754 |  |  |
| Figure S2h | Control | Stress | *F _(19,25)_* =1.13 | *p =* 0.8007 |
|  | *p* > 0.1000 | *p* > 0.1000 |  |  |
| Figure S3a relative length | Control | Stress | *F _(5,5)_* =15.15 | *** p =* 0.0097 |
|  | *p* > 0.1000 | *p* > 0.1000 |  |  |
| Figure S3a relative area | Control | Stress | *F _(5,5)_* =22.99 | *** p =* 0.0037 |
|  | *p* > 0.1000 | *p* > 0.1000 |  |  |
| Figure S3a diameter | Control | Stress | *F _(17,17)_* =5.194 | *** p =* 0.0015 |
|  | *p* > 0.1000 | *p* = 0.3122 |  |  |
| Figure S3b length | Control | Stress | / | / |
|  | ** p* = 0.0272 | ** p* = 0.0128 |  |  |
| Figure S3b area | Control | Stress | / | / |
|  | ** p* = 0.0278 | *p* = 0.0738 |  |  |
| Figure S3b diameter | Control | Stress | *F _(17,17)_* =1.687 | *p =* 0.2909 |
|  | *p* > 0.1000 | *p* > 0.1000 |  |  |
| Figure S3c length | Control | Stress | *F _(5,5)_* =2.323 | *p =* 0.3765 |
|  | *p* > 0.1000 | *p* > 0.1000 |  |  |
| Figure S3c area | Control | Stress | *F _(5,5)_* =2.661 | *p =* 0.3064 |
|  | *p* > 0.1000 | *p* > 0.1000 |  |  |
| Figure S3c diameter | Control | Stress | *F _(17,17)_* =1.093 | *p =* 0.8569 |
|  | *p* > 0.1000 | *p* > 0.1000 |  |  |
| Figure S3d | Control | Stress | / | / |
|  | ** p* = 0.0068 | *p* > 0.1000 |  |  |
| Figure S3e | Control | Stress | *F _(5,5)_* =3.572 | *p =* 0.1887 |
|  | *p* > 0.1000 | *p* > 0.1000 |  |  |
| Figure S4e | Control | Stress | *F _(4,5)_* =3.954 | *p =* 0.1640 |
|  | *p* > 0.1000 | *p* > 0.1000 |  |  |
| Figure S4f Left | Control | Stress | *F _(4,5)_* =1.764 | *p =* 0.5455 |
|  | *p* > 0.1000 | *p* > 0.1000 |  |  |
| Figure S4f Right | Control | Stress | *F _(4,5)_* =1.046 | *p =* 0.9928 |
|  | *p* > 0.1000 | *p* > 0.1000 |  |  |

**Table S4.** Data of mean, SEM and N for each group.

| **Figures.** | | **Ctrl/(Control)** | | **Stress/(Corticosterone)** | | **N** |
| --- | --- | --- | --- | --- | --- | --- |
|  |  | **mean** | **SEM** | **mean** | **SEM** |  |
| Figure. 3b | length | 17.96 | 0.04958 | 16.02 | 0.1523 | 6 |
|  | area | 46.4 | 1.077 | 39.63 | 0.7953 | 6 |
| Figure. 3c | length | 10.35 | 0.08989 | 8.903 | 0.2135 | 6 |
|  | area | 27 | 1.021 | 21.92 | 0.4693 | 6 |
| Figure. 3d | length | 7.604 | 0.05379 | 7.117 | 0.2221 | 6 |
|  | area | 19.4 | 0.5702 | 17.72 | 0.6044 | 6 |
| Figure. 3e |  | 45.99 | 0.8877 | 46.06 | 0.7516 | 72 |
| Figure. 3g | length | 3.915 | 0.2451 | 3.247 | 0.2053 | 12 (left and right hippocampi from 6 mice) |
|  | area | 18.29 | 1.162 | 15.11 | 0.9180 | 12 (same as above) |
| Figure. 3h | length | 2.036 | 0.1178 | 1.612 | 0.1043 | 12 (same as above) |
|  | area | 9.526 | 0.5881 | 7.374 | 0.3837 | 12 (same as above) |
| Figure. 3i | length | 1.880 | 0.1617 | 1.633 | 0.1567 | 12 (same as above) |
|  | area | 8.759 | 0.8727 | 7.729 | 0.8241 | 12 (same as above) |
| Figure. 3j |  | 39.23 | 1.189 | 37.07 | 1.252 | 72 |
| Figure. 3l |  | 1.226 | 0.08167 | 1.192 | 0.05521 | 6 |
| Figure. 3m |  | 1.671 | 0.1852 | 1.894 | 0.149 | 6 |
| Figure. 3n |  | 35.06 | 5.147 | 49.99 | 5.258 | 18 |
| Figure. 3p |  | 0.122 | 0.02083 | 0.075 | 0.005 | n1=5  n2=6 |
| Figure. 4b |  | 0.1788 | 0.04886 | 0.3457 | 0.04076 | 7 |
| Figure. 4c |  | 0.7398 | 0.02529 | 0.7935 | 0.1020 | 7 |
| Figure. 4d |  | 0.04710 | 0.004654 | 0.03578 | 0.005509 | 7 |
| Figure. 4f |  | 1.397 | 0.3606 | 1.192 | 0.2044 | 7 |
| Figure. 4g |  | 0.3303 | 0.04535 | 0.1499 | 0.02268 | 7 |
| Figure. 4h |  | 1.527 | 0.2222 | 0.8987 | 0.09201 | 7 |
| Figure. 4i |  | 0.6217 | 0.04595 | 0.9184 | 0.09391 | 7 |
| Figure. 4l |  | 0.6281 | 0.03558 | 1.567 | 0.4210 | 6 |
| Figure S2b | BL | 100 | 3.9495 | 100 | 3.949 | n1=10  n2=14 |
|  | Day 7 | 111.055 | 5.2133 | 101.931 | 3.458 |  |
|  | Day 14 | 111.353 | 3.4607 | 101.164 | 3.374 |  |
|  | Day 21 | 104.285 | 4.1524 | 86.675 | 2.649 |  |
| Figure S2c |  | 176.9 | 6.081 | 202.4 | 3.727 | n1=20  n2=26 |
| Figure S2d | Left | 2544 | 283.6 | 3420 | 355.0 | n1=20  n2=26 |
|  | right | 5.359 | 0.6038 | 3.552 | 0.3053 | n1=20  n2=26 |
| Figure S2e | BL | 0.000 | 0.000 | 0.000 | 0.000 | n1=20  n2=26 |
|  | 4 | -0.055 | 0.101 | -0.758 | 0.208 |  |
|  | 8 | -0.025 | 0.167 | -0.842 | 0.215 |  |
|  | 12 | 0.175 | 0.206 | -0.692 | 0.228 |  |
|  | 16 | 0.260 | 0.251 | -0.673 | 0.202 |  |
|  | 20 | -0.120 | 0.163 | -1.115 | 0.226 |  |
|  | 24 | -0.040 | 0.200 | -0.962 | 0.273 |  |
| Figure S2f |  | 45.09 | 5.589 | 83.46 | 13.11 | n1=8  n2=13 |
| Figure S2g |  | 0.1162 | 0.004621 | 0.06763 | 0.004301 | n1=20  n2=26 |
| Figure S2h |  | 0.3181 | 0.01353 | 0.3133 | 0.02630 | n1=10  n2=14 |
| Figure S3a | Relative length | 9.096 | 0.4524 | 5.313 | 0.1162 | 6 |
|  | Relative area | 4.932 | 0.4251 | 3.049 | 0.08866 | 6 |
|  | Diameter | 29.06 | 0.8409 | 28.39 | 0.3690 | 18 |
| Figure S3b | Length | 1.418 | 0.08806 | 1.392 | 0.09257 | 6 |
|  | Area | 5.211 | 1.071 | 6.066 | 1.449 | 6 |
|  | Diameter | 16.27 | 0.4289 | 16.95 | 0.3302 | 18 |
| Figure S3c | Length | 6.350 | 0.5044 | 7.302 | 0.7686 | 6 |
|  | Area | 3.553 | 0.4005 | 4.118 | 0.6533 | 6 |
|  | Diameter | 22.58 | 0.8667 | 21.00 | 0.8290 | 18 |
| Figure S3d |  | 2.374 | 0.1721 | 2.013 | 0.1542 | 6 |
| Figure S3e |  | 2.166 | 0.0682 | 2.182 | 0.1289 | 6 |
| Figure S4d | BL | 0.000 | 0.000 | 0.000 | 0.000 | n1=5  n2=6 |
|  | 4 | 0.080 | 0.380 | -0.117 | 0.214 |  |
|  | 8 | -0.180 | 0.097 | -0.983 | 0.204 |  |
|  | 12 | 0.860 | 0.311 | -0.467 | 0.242 |  |
|  | 16 | 0.820 | 0.343 | -0.300 | 0.273 |  |
| Figure S4e |  | 124 | 11.49 | 154.7 | 5.277 | n1=5  n2=6 |
| Figure S4f | Left | 1777 | 173.7 | 1667 | 119.4 | n1=5  n2=6 |
|  | Right | 5.16 | 0.4806 | 3.311 | 0.4487 | n1=5  n2=6 |

**Movie S1**

Provided as a separate file (Movie S1.mp4). LYVE1 signals in whole brain (length: 30 s). A 3D reconstruction of a whole mouse brain showing LYVE1 immunostaining signal detected on the meninges of the brain, especially around the superior sagittal sinus area. Optical sectioning of the whole brain showed that the LYVE1 signal can be observed deep inside the brain.

**Movie S2**

Provided as a separate file (Movie S2.mp4). LYVE1 and CD34 (blood vessels markers) signals in brain (length: 30 s). A 3D reconstruction of part of mouse brain showing LYVE1 and CD34 signals do not merge and that the blood vessels network is much more complicated than that of lymphatic vessels.

**Movie S3**

Provided as a separate file (Movie S3.mp4). Split brain region by using Surface algorithm (length: 54 s). Brain regions with the strongest LYVE1 signals were delineated. The 3D structure of cortex, hindbrain, cerebellum and hippocampus was constructed.

**Movie S4**

Provided as a separate file (Movie S4.mp4). Lymphatic vessels in hippocampus (length: 41 s). In the space separating the hippocampus and midbrain, the outer lymphatic network extensively supplied both the hippocampus and thalamus with lymphatic vessels. The lymphatic vessels within the hippocampus extended dorsolaterally.

**Movie S5**

Provided as a separate file (Movie S5.mp4). Lymphatic vessels in hippocampus of Control and Stress mice (light sheet microscopy, length: 21 s). Representative 3D reconstruction of the lymphatic vessels network within the hippocampus of mice from the stress and control groups. The structure was constructed from whole-brain imaging by light sheet microscopy.

**Movie S6**

Provided as a separate file (Movie S6.mp4). Lymphatic vessels in hippocampus of Control and Stress mice (confocal microscopy, length: 16 s). Representative 3D reconstruction of the lymphatic vessels network within the hippocampus of mice from the stress and control groups. The structure was constructed from thick brain section imaging by confocal microscopy.
